# Elucidating the mechano-molecular dynamics of TRAP activity using CRISPR/Cas9 mediated fluorescent reporter mice

**DOI:** 10.1016/j.heliyon.2024.e32949

**Published:** 2024-06-13

**Authors:** Dilara Yılmaz, Francisco C. Marques, Yannick Fischer, Sandra Zimmermann, Gaonhae Hwang, Penny R. Atkins, Neashan Mathavan, Amit Singh, Pedro P.C. de Souza, Gisela A. Kuhn, Esther Wehrle, Ralph Müller

**Affiliations:** a Institute for Biomechanics, ETH Zürich, Zürich, Switzerland; b Department of Orthopaedics, University of Utah, 590 Wakara Way, Salt Lake City, USA; c Innovation in Biomaterials Laboratory, School of Dentistry, Federal University of Goiás, Goiânia, Brazil; d AO Research Institute Davos, Davos Platz, Switzerland

**Keywords:** Mechanosensitivity, CRISPR/Cas9 mediated reporter mice, Fluorescent osteoclasts and osteoblasts, TRAP deficiency, Trabecular bone remodeling, Micro-computed tomography, Micro-finite element analysis, In vivo loading, Bone remodeling

## Abstract

Osteoclasts are essential for bone remodeling by adapting their resorptive activity in response to their mechanical *in vivo* environment. However, the molecular mechanisms underlying this process remain unclear. Here, we demonstrated the role of tartrate-resistant acid phosphatase (TRAP, Acp5), a key enzyme secreted by osteoclasts, in bone remodeling and mechanosensitivity. Using CRISPR/Cas9 reporter mice, we demonstrated bone cell reporter (BCR^Ibsp/Acp5^) mice feature fluorescent TRAP-deficient osteoclasts and examined their activity during mechanically driven trabecular bone remodeling. Although BCR^Ibsp/Acp5^ mice exhibited trabecular bone impairments and reduced resorption capacity *in vitro*, RNA sequencing revealed unchanged levels of key osteoclast-associated genes such as C*tsk, Mmp9,* and *Calcr*. These findings, in conjunction with serum carboxy-terminal collagen crosslinks (CTX) and *in vivo* mechanical loading outcomes collectively indicated an unaltered bone resorption capacity of osteoclasts *in vivo*. Furthermore, we demonstrated similar mechanoregulation during trabecular bone remodeling in BCR^Ibsp/Acp5^ and wild-type (WT) mice. Hence, this study provides valuable insights into the dynamics of TRAP activity in the context of bone remodeling and mechanosensation.

## Highlights

• BCR^Ibsp/Acp5^ mice feature osteoclasts that are TRAP-deficient and fluorescent.
• BCR^Ibsp/Acp5^ mice display trabecular deformities and reduced resorption levels.
• TRAP deficiency does not influence osteoclast-associated gene expression levels.
• *In vivo* mechanosensitivity is maintained in TRAP-deficient BCR^Ibsp/Acp5^ mice.

## Graphical abstract

**Figure:**
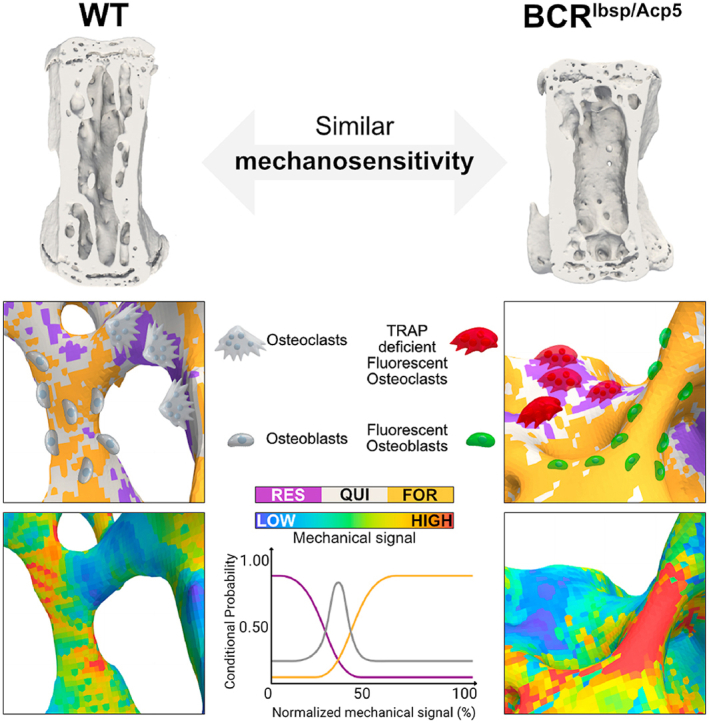

## 1 Introduction

The skeleton undergoes continuous remodeling as old bone is removed and new bone is formed [[1], [2], [3]]. This intricate process of bone remodeling involves highly coordinated activity of bone-resorbing osteoclasts and bone-forming osteoblasts both of which are influenced by mechanical strains in the mechanical *in vivo* environment of these cells [4]. Bone formation has been associated with regions subjected to high strains, while bone resorption was more frequently observed in regions with low strains [[5], [6], [7], [8]]. Notably, this effect is more evident in trabecular bone, that is more sensitive to mechanical loading due to its higher metabolic activity and mechanical heterogeneity compared to cortical bone [9]. Furthermore, bone resorption was shown to be more tightly regulated by mechanical loading in comparison to bone formation, thus highlighting the key role of osteoclasts in bone remodeling and mediating bone loss [10]. Dysregulation of the function of osteoclasts has been linked to numerous diseases, including osteoporosis, osteopetrosis, and rheumatoid arthritis [[11], [12], [13]]. There has been limited reporting on the impact of mechanical stimuli on osteoclasts, and the precise mechanism underlying their response to mechanical stress is yet to be fully elucidated.

Tartrate-resistant acid phosphatase (TRAP; Acp5) is a pivotal enzyme predominantly found in osteoclasts, macrophages, and dendritic cells [14]. TRAP encodes for two distinct isoforms (TRAP 5a and TRAP 5b). TRAP 5a is a peptide loop protein that interacts with the active site of the enzyme to inhibit phosphatase activity. The loop region undergoes proteolytic cleavage resulting in the formation of dimeric TRAP 5b with enhanced phosphatase activity [15]. While TRAP 5a is secreted from macrophages and dendritic cells, TRAP 5b is transported through osteoclasts by transcytosis. Therefore, it is released into the bloodstream during bone resorption and serves as a marker for osteoclast activity [3,16]. Additionally, TRAP 5b has been suggested to regulate osteoclast migration through dephosphorylation of osteopontin (OPN) [17]. Both of these isoforms of TRAP have been shown to be secreted by differentiating osteoclasts and are correlated to C-terminal type I collagen cross-linked peptide (CTX). Increased TRAP 5b release from bone suggests a link to osteoclast resorptive activity, and a peak in the TRAP 5b/5a ratio coincides with a rapid release of CTX [18]. Notably, the inactivation of the human *TRAP* gene (*Acp5*) results in the hyperphosphorylation of *OPN* [19], thus further emphasizing the intricate relationship between TRAP isoforms and the modulation of cellular processes. TRAP also plays an important role in facilitating osteoclast migration to bone resorption sites. Once bone remodeling is initiated, TRAP level increases in the plasma and initiates crucial processes such as osteoclast differentiation, activation, and proliferation [20,21].

In TRAP knock-out studies, the absence of TRAP led to widened and disorganized growth plates, skeletal shortening and deformity, and increased mineralization of bones linked to mild osteopetrosis due to dysfunctional osteoclast activity [2,20]. Thus, osteoclasts are important for remodeling, responding to mechanical signals, and secreting essential markers for resorption, such as TRAP, whose dysregulation is a precursor to significant bone diseases.

Additionally, mouse loading models have enabled controlled experimental conditions to investigate bone adaptation in response to physiological and supraphysiological mechanical loading [[22], [23], [24], [25]]. In combination with imaging techniques with improved spatial and temporal resolution, it is possible to monitor bone remodeling *in vivo* at tissue and cellular levels. Particularly, in-depth characterization of mechanical *in vivo* environment during bone remodeling by correlating time-lapsed *in vivo* micro-computed tomography (micro-CT) measurements with micro-finite element simulations (micro-FE). By registering consecutive micro-CT time points, remodeling sites can be accurately identified and associated with local mechanical signals computed with micro-FE [8,26]. In this regard, the role of TRAP in bone mechanoregulation, when considering the influence of supraphysiological mechanical loading on trabecular bone adaptation, is yet to be elucidated. Understanding osteoclast biology and the critical function of TRAP in bone turnover is essential, as it guides the development of TRAP inhibitors and potential therapeutics for osteoclast-related bone abnormalities.

Here, we provide insight into the function of TRAP in mechanically driven trabecular bone adaptation and mechanosensitivity using an *in vivo* loading mouse model. We used the BCR^Ibsp/Acp5^ mouse model, generated via CRISPR/Cas9 technology, which features osteoclasts that are TRAP-deficient and fluorescently marked with mCherry (*Acp5-mCherry*). Simultaneously, the osteoblasts remain intact and are also fluorescently labeled with GFP (*Ibsp-eGFP*). This dual-tagging approach enables us to track osteoblasts and osteoclasts with TRAP-deficiency both *in vivo* and *in vitro* effectively. Additionally, BCR^Ibsp/WT^ mice, which express only *Ibsp-eGFP*, were used as internal controls. By combining *in vivo* loading and time-lapsed micro-CT with *in vitro* cell assays and RNA sequencing, we further demonstrated trabecular bone adaptation at the molecular level in BCR^Ibsp/Acp5^ mice. Moreover, we used *in silico* micro-FE simulations to characterize trabecular mechanoregulation at sites of bone remodeling in our TRAP-deficient mouse model. Our findings revealed that mechanosensitivity is maintained in BCR^Ibsp/Acp5^ mice despite exhibiting a deteriorated trabecular bone structure and reduced osteoclast function.

Overall, this study was motivated by the lack of understanding of the function of TRAP in bone remodeling and mechanosensation. Utilizing CRISPR/Cas9 technology, we have created a reporter mouse model characterized by TRAP deficiency, with osteoclasts and osteoblasts fluorescently labeled, which allows for the tracking of these cells. Our comprehensive methodology includes the characterization of both cell types through *in vivo, in vitro*, and *in silico* approaches aimed to elucidate the dynamics of the TRAP activity in bone remodeling. Insights gained from this study could lead to new treatments targeting diseases stemming from osteoclast abnormalities.

## 2 Results

### 2.1 *In vivo* identification and characterization of fluorescent bone cells in BCR^Ibsp/Acp5^ mice

**Fig. 1:**
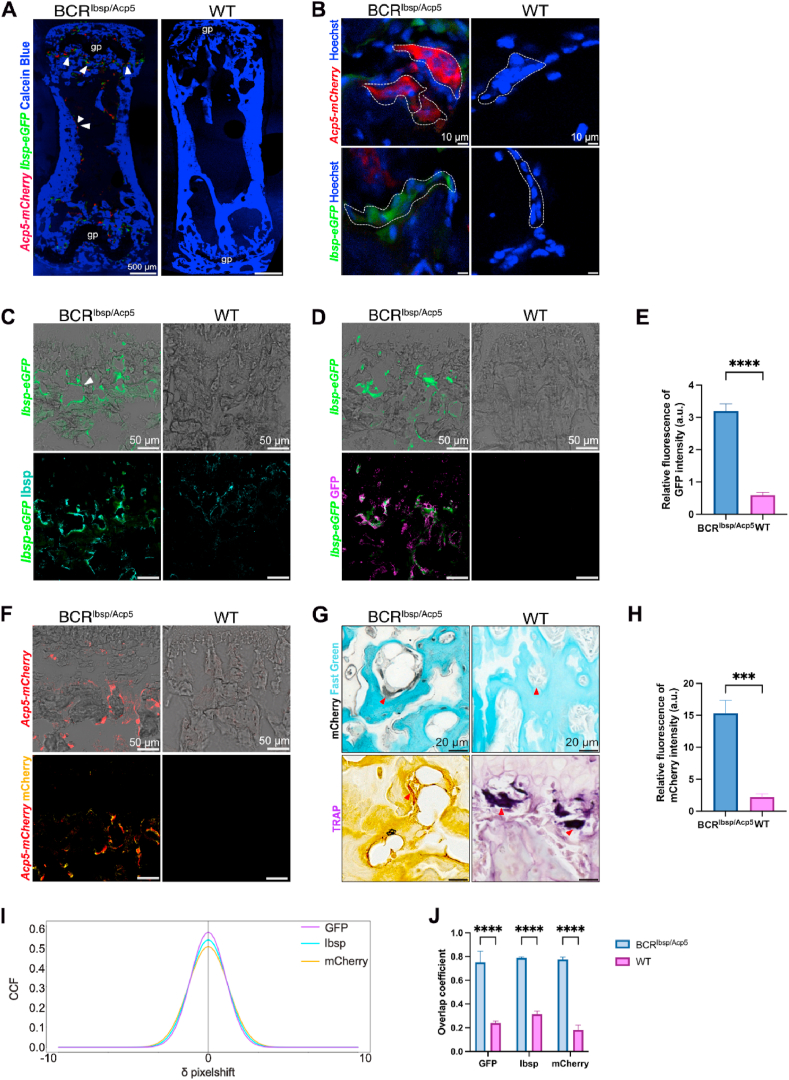
Characterization of fluorescent bone cells of BCR^Ibsp/Acp5^ mice *in vivo*. A) Representative tile scan confocal images of the caudal vertebra showing mCherry (*Acp5-mCherry,* red), GFP (*Ibsp-eGFP*, green), and Calcein Blue (mineral, blue) staining in 5-week-old BCR^Ibsp/Acp5^ and WT mice. B) High-magnification images showing fluorescent signals of osteoclasts (*Acp5-mCherry*, red) and osteoblasts (*Ibsp-eGFP*, green) stained with Hoechst (nuclear, blue) in 5-week-old BCR^Ibsp/Acp5^ and WT mice. C-D) Colocalization of GFP (*Ibsp-eGFP*, green) with GFP and Ibsp immunostainings. GFP signal (*Ibsp-eGFP*, green) is shown on bright-field images for each staining. C) Representative maximum intensity projections images of BCR^Ibsp/Acp5^ and WT vertebra stained for GFP (purple) and D) Ibsp (cyan). E) Relative fluorescence intensity (a.u.) of GFP (*Ibsp-eGFP*) signals of BCR^Ibsp/Acp5^ and WT vertebra sections. F) Colocalization of mCherry (*Acp5-mCherry*, red) with mCherry immunostaining (yellow). mCherry (*Acp5-mCherry,* red) signal is shown on bright-field images. G) mCherry immunostaining co-stained with fast green (turquoise) and TRAP (purple) staining in BCR^Ibsp/Acp5^ and WT mice. Arrowheads indicate osteoclasts. H) Relative fluorescence intensity (a.u.) of mCherry (*Acp5-mCherry*) signals of BCR^Ibsp/Acp5^ and WT vertebra sections. I) Quantitative Van Steensel's analyses and overlap colocalization coefficient. Data represent mean ± s.d., (n = 3 male and/or female mice/group), *< 0.05, **< 0.01, ***< 0.001 according to two-way ANOVA with Sidak's comparison. (For interpretation of the references to colour in this figure legend, the reader is referred to the Web version of this article.)

To identify the characteristics and morphology of the fluorescently tagged cells *in vivo,* we performed cell-specific immunostaining in caudal vertebrae and femurs of BCR^Ibsp/Acp5^ mice and compared them to WT controls at ages between 5 and 25 weeks. While the expression profile of labeled TRAP is broader and encompasses not only osteoclasts but also macrophages and immune cell types, the term “fluorescent osteoblast” similarly denotes not only osteoblasts but also osteoblastic cell types such as lining cells. Fluorescently labeled osteoblasts and osteoclasts with TRAP-deficiency were mainly located in the trabecular bone surfaces, around the growth plate, and along the cortex of BCR^Ibsp/Acp5^ mice whereas WT mice lacked both signals in caudal vertebrae and femurs (Fig. 1A). Calcein Blue stained the mineralized bone surfaces from the caudal vertebrae to outline the bone structure. Multinucleated osteoclasts with TRAP-deficiency expressing mCherry, and mononucleated lining cells and osteoblasts expressing GFP around the bone surface were identified with Hoechst staining (Fig. 1B). TRAP staining revealed the absence of TRAP expression in BCR^Ibsp/Acp5^ mice compared to that in WT mice, and mCherry immunostaining confirmed the mCherry signal only in BCR^Ibsp/Acp5^ mice (Fig. 1F and G). Moreover, IBSP, GFP, and mCherry immunostainings validated the co-localization of GFP and mCherry signals with the respective stainings and accentuated the specificity of the tagged cells (Fig. 1C–D, F). The relative fluorescence intensities of GFP and mCherry further verified the expression of mCherry and GFP signals from BCR^Ibsp/Acp5^ in contrast to WT mice lacking fluorescent protein expressions (Fig. 1E–H). Additionally, Van Steensel's co-localization analysis quantitatively measured the overlap between fluorescent signals with their respective immunostainings in BCR^Ibsp/Acp5^ mice. The complete colocalization was indicated by a peak at δx = 0 and a bell-shaped curve [27]. Although differences in ﬂuorescence intensity can decrease the height of this curve, the peak remains at δx = 0. The specific overlap between GFP and mCherry signals and their corresponding immunostainings was confirmed for BCR^Ibsp/Acp5^ in comparison to WT mice through the pronounced bell-shaped curve peak at δx = 0, with a mean cross-correlation (CCF) of 0.55 ± 0.03 in GFP, 0.50 ± 0.07 in Ibsp, 0.45 ± 0.09 in mCherry (Fig. 1I and J). Overall, these data confirmed the presence and specificity of GFP-tagged osteoblasts and mCherry-tagged osteoclasts with TRAP-deficiency from BCR^Ibsp/Acp5^ mice *in vivo*.

Furthermore, micro-CT analysis of the caudal vertebrae in BCR^Ibsp/Acp5^ mice demonstrated differences in bone structure between TRAP-deficient mice and controls. Trabecular bone volume fraction (BV/TV) of BCR^Ibsp/Acp5^ mice was reduced on average −30 % compared to BCR^Ibsp/WT^ and −42 % on average compared to WT mice. In addition, trabecular number in TRAP-deficient BCR^Ibsp/Acp5^ mice was decreased compared to BCR^Ibsp/WT^ and WT mice (−5% and −6% on average, respectively), followed by increased trabecular space (+8 % average compared to BCR^Ibsp/WT^ and +9 % compared to WT mice), indicating notably impaired trabecular bone in BCR^Ibsp/Acp5^ mice (Figure S1A- C, Supplementary Table 1). BCR^Ibsp/Acp5^ mice also displayed a significant shortening of the vertebra, as indicated by longitudinal 3D images comparing the vertebra length of BCR^Ibsp/Acp5^ mice with BCR^Ibsp/WT^ and WT mice (on average, 21 % and 20 % reduction, respectively) (Figs. S1A and C, Supplementary Table 1). However, no clear differences were found between groups regarding cortical bone development, as shown by 3D cross-sectional images (Fig. S1B). Supporting this data, there were no significant differences between BCR^Ibsp/Acp5^, BCR^Ibsp/WT^, and WT mice in cortical bone parameters, including cortical bone area, total cortical area, cortical area fraction, and cortical thickness (Fig. S1E). Similar skeletal deformities observed in vertebra were also noted in femurs of BCR^Ibsp/Acp5^ mice in comparison to BCR^Ibsp/WT^ and WT mice (Fig. S2A, Supplementary Table 2). 3D reconstructed micro-CT images showed significant shortening of femurs with a 12 % reduction for both controls and indicated trabecular bone impairments in BCR^Ibsp/Acp5^ mice compared to BCR^Ibsp/WT^ and WT (Figs. S2A–B, Supplementary Table 2). In contrast to the vertebra, trabecular BV/TV in the femurs significantly increased in BCR^Ibsp/Acp5^ mice compared to other groups (+74 % in comparison to BCR^Ibsp/WT^ and +70 % compared to WT), while no change was observed in trabecular number and separation (Fig. S2B). As in vertebra, cortical parameters were similar for all groups in femurs, with cortical thickness, cortical bone area, and total cortical area showing no notable differences; however, cortical area fraction was found to be lower compared to WT (−13 %) but not to BCR^Ibsp/WT^ (Fig. S2C). Taken together, this data suggests that BCR^Ibsp/Acp5^ mice display morphological deformities in the trabecula and the skeleton**.**

### 2.2 Osteoclasts with TRAP deficiency display an altered morphology and function

**Fig. 2:**
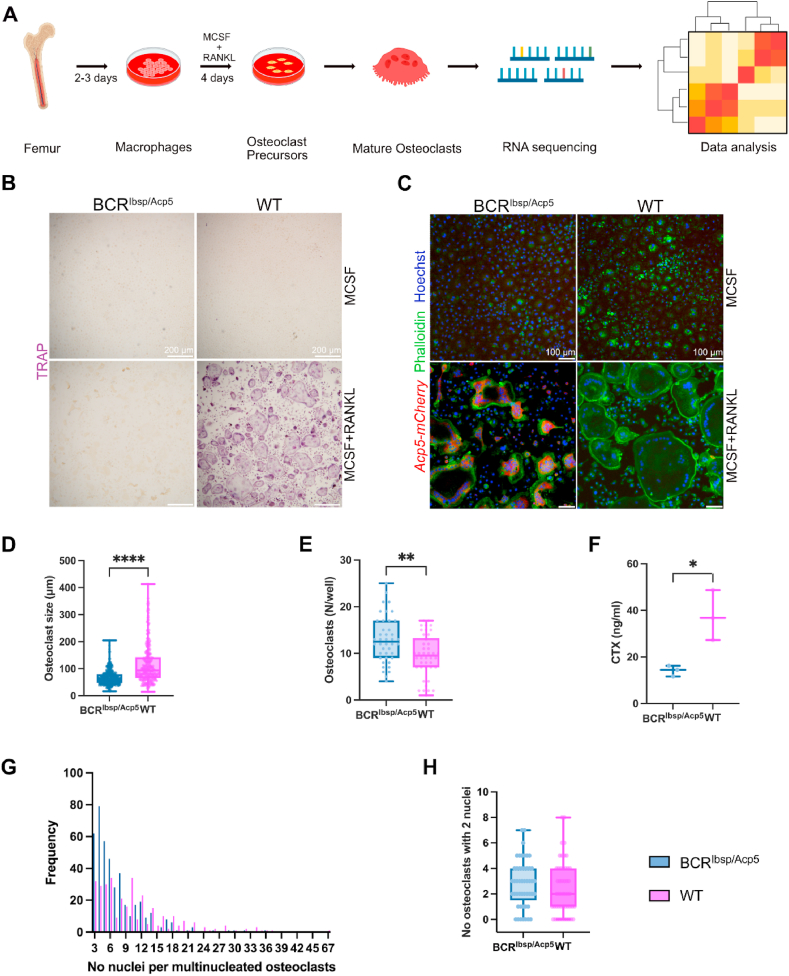
Cellular and functional properties of TRAP-deficient osteoclasts. A) Schematic showing the method for isolating and culturing primary osteoclasts from the femurs of BCR^Ibsp/Acp5^ and WT mice at 6 weeks old. B) TRAP (purple) staining showed TRAP ^+^ osteoclasts isolated from BCR^Ibsp/Acp5^ and WT mice cultured in the presence of RANKL (created with Biorender) C) Representative images of mCherry (*Acp5-mCherry*, red), Phalloidin (F-Actin, green), and Hoechst (nucleus, blue) staining in osteoclasts isolated from BCR^Ibsp/Acp5^ and WT mice cultured in the presence of RANKL. D) Size of Phalloidin and Hoechst positive multinucleated cells generated from macrophages isolated from BCR^Ibsp/Acp5^ and WT mice in the presence of RANKL. E) Number of Phalloidin and Hoechst positive multinucleated cells generated from macrophages isolated from BCR^Ibsp/Acp5^ and WT mice in the presence of RANKL. F) Measured CTX protein levels of osteoclasts derived from BCR^Ibsp/Acp5^ and WT mice cultured on the bone disks in the presence of RANKL. G) Number of nuclei per multinucleated osteoclasts (>2 nuclei) from BCR^Ibsp/Acp5^ and WT mice cultured in the presence of RANKL. Data represent mean ± s.d. (n = 3 male mice/group), ****< 0.0001 according to Kolmogorov-Smirnov test. H) Number of nuclei per osteoclast with two nuclei from BCR^Ibsp/Acp5^ and WT mice cultured in the presence of RANKL. Data represent mean ± s.d. (n = 3 male mice/group), *< 0.05, **< 0.01, ***< 0.001 according to two-tailed student t-test. (For interpretation of the references to colour in this figure legend, the reader is referred to the Web version of this article.)

Next, we isolated bone marrow macrophages from BCR^Ibsp/Acp5^ and WT mice and cultured them *in vitro* to study the features of osteoclats with TRAP-deficiency(Fig. 2A). The same number of bone marrow macrophages was initially seeded for BCR^Ibsp/Acp5^ and WT mice to differentiate into osteoclasts in the presence of receptor activator of nuclear factor kappa B ligand (RANKL). Macrophages (cells treated with macrophage colony-stimulating factor (MCSF) and without RANKL) were used as the internal controls (Fig. 2B and C). Similar to the *in vivo* data, TRAP staining confirmed the expression of TRAP ^+^ osteoclasts in WT mice but not in osteoclasts from BCR^Ibsp/Acp5^ mice (Fig. 2B). Nonetheless, osteoclasts with TRAP deficiency from BCR^Ibsp/Acp5^ mice were positive for *Acp5-mCherry*, while this signal was absent in osteoclasts from WT mice, as expected (Fig. 2C). Formation of F-actin rings that are a characteristic feature of mature osteoclasts was observed by Phalloidin-Hoechst staining in both groups (Fig. 2C). Multinucleated (>2 nuclei) osteoclasts were identified in BCR^Ibsp/Acp5^ mice and were similar to WT mice; however, these multinucleated osteoclasts in TRAP-deficient mice were smaller in size, irregular in shape (67.5 mm ± 27.0 for BCR^Ibsp/Acp5^; 116.3 mm ± 73.3 for WT, p < 0.0001) and more abundant (12.9 ± 4.9 for BCR^Ibsp/Acp5^; 9.5 ± 4.5 for WT, p = 0.0014) as presented in Fig. 2D and E. Moreover, osteoclasts in BCR^Ibsp/Acp5^ mice not only displayed irregular shapes and small sizes but also exhibited lower nuclear count compared to those of WT osteoclasts (Fig. 2G). Most of the observed osteoclasts in BCR^Ibsp/Acp5^ mice contained 3–4 nuclei and the osteoclast with the highest nuclei count was 29 nuclei. In contrast, osteoclasts in WT mice, displayed an average of 10 nuclei, whereas the largest osteoclasts contained up to 47 nuclei (Fig. 2G). Differences in the number of nuclei per osteoclasts between the groups were confirmed to be statistically significant using the Kolmogorov-Smirnov test (p < 0.0000.1) (Fig. 2D). However, there was no discernible difference in the number of pre-osteoclasts containing two nuclei between BCR^Ibsp/Acp5^ and WT mice (Fig. 2H). These data suggested that osteoclasts from BCR^Ibsp/Acp5^ mice are unable to form large highly nucleated osteoclasts, thus indicating a compromised fusion process, whereas the pre-osteoclast formation remains unaffected. Additionally, when the cells were cultured on bone disks, the concentration of CTX, a serum marker indicating the bone resorption, was decreased by 80 % in osteoclasts from BCR^Ibsp/Acp5^ mice compared to those from WT, thus illustrating that the resorption capacity of osteoclasts was reduced in BCR^Ibsp/Acp5^ mice (Fig. 2F).

**Fig. 3:**
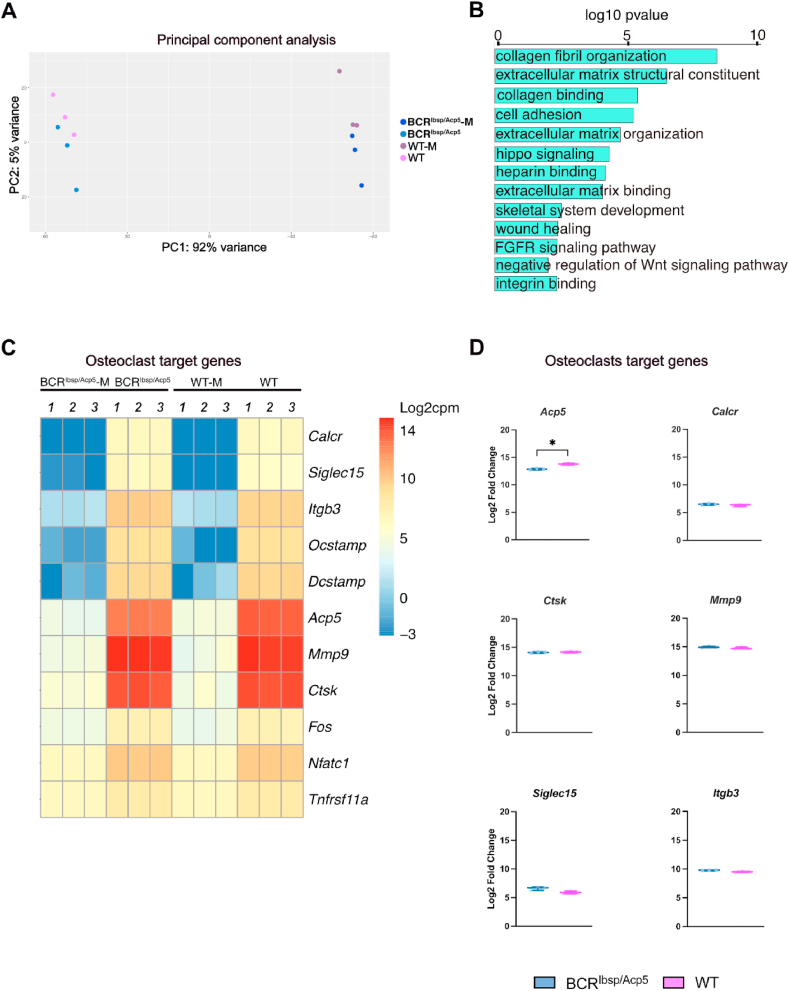
Transcriptional landscape and gene expression pattern of osteoclasts from BCR^Ibsp/acp5^ mice. A) Principal component analysis of RNA-sequencing data using the top 500 most variable genes across the samples. The first component shows 92 % variance (PC1), and the second component (PC2) shows 5 % variance within sample groups. B) Downregulated signaling pathway components between primary osteoclasts derived from BCR^Ibsp/Acp5^ and WT mice (in the presence of RANKL). C) Heatmap of osteoclasts target genes in primary osteoclast derived from BCR^Ibsp/Acp5^ and WT mice (in the presence of RANKL) and macrophages (in the presence of MCSF without RANKL). Color intensity indicates normalized log2 (cpm) expression values. D) Differentially regulated osteoclasts target genes (*Acp5, Calcr, Ctsk, Mmp9, Siglec15, Itgb3*) in BCR^Ibsp/Acp5^ vs. WT mice. Data represent the normalized log_2_ (cpm) of RNA seq data, mean ± s.d. (n = 3 male mice/group), *< 0.05, **< 0.01, ***p< 0.001 according to Mann-Whitney test. (For interpretation of the references to colour in this figure legend, the reader is referred to the Web version of this article.)

To further gain insight into the characteristics of osteoclasts with TRAP-deficiency, we performed bulk RNA sequencing (RNA-seq) analysis of cells obtained from BCR^Ibsp/Acp5^ and WT mice that were isolated and cultured *in vitro*. Principal component analysis (PCA) of the RNA-seq data indicated a 92 % variation between BCR^Ibsp/Acp5^ and WT samples, that fell into two groups: macrophages (BCR^Ibsp/Acp5^-M, WT-M) and osteoclasts (BCR^Ibsp/Acp5^, WT). Within each group, gene expression levels exhibited less variations (Fig. 3A). Gene set enrichment analysis revealed a downregulation in BCR^Ibsp/Acp5^ mice of essential signaling pathways promoting growth and proliferation, such as Hippo, fibroblast growth factor receptors (FGFR), and negative regulators of the Wnt signaling pathway (Fig. 3B). Consistent with immunostaining results, *TRAP* (*Acp5*) was downregulated in osteoclasts from BCR^Ibsp/Acp5^ mice, whereas genes important for osteoclastogenesis such as *Ctsk*, *Calcr*, *Nfatc1*, and *Tnfrsf11a*, exhibited the same degree of expression in BCR^Ibsp/Acp5^ and WT mouse osteoclasts (Fig. 3C and D). Taken together, these results indicated that reduced TRAP activity did not alter the expression of other essential osteoclast-associated genes (Fig. 3D).

### 2.3 BCR^Ibsp/Acp5^ mice exhibit a similar response to *in vivo* mechanical loading as WT mice

**Fig. 4:**
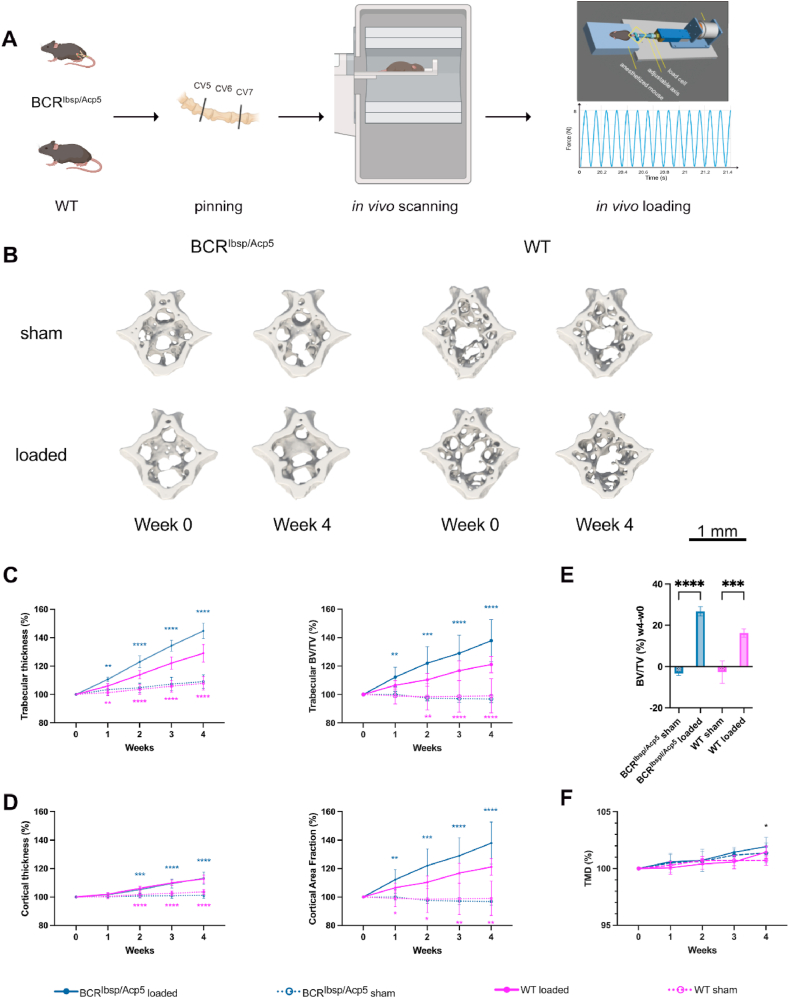
Micro-CT-based evaluation of static bone parameters upon cyclic mechanical loading. A) Schematic indicating the *in vivo* tail loading setup: the application of force is controlled through two pins inserted in the neighboring vertebrae (CV5 and CV7) connected to a mechanical loading device, and the 6th caudal vertebra (CV6) was either cyclically loaded with a force of 8 N or sham-loaded (0 N) (created with Biorender). All mice were scanned weekly using micro-CT for 4 weeks. B) Representative micro-CT images from the sham and loaded groups of BCR^Ibsp/Acp5^ and WT mice were taken at two-time points: the initial time point (week 0) and the final time point (week 4) during the 4-week loading period. *In vivo* micro-CT scans were conducted to capture the changes in bone microstructure. For loading groups, thickening of the trabecular structure can be observed, while in the sham groups, minimal changes in the bone structure between two time points can be observed. C-D) Changes in the structural bone morphometric parameters over the 4-week tail loading: trabecular thickness, trabecular bone volume fraction (BV/TV), cortical thickness, and cortical area fraction E) Change in bone volume fraction from week 4 to week 0 (BV/TV week 4/week 0). F) Changes in the tissue mineral density (TMD) over the 4-week tail loading. All data were normalized to the first time point and presented as percentages. Data represent mean ± s.d. (n = 9–10 female mice/group), *< 0.05, **< 0.01, ***< 0.001 according to two-way ANOVA with Dunnett comparison test.

Next, we investigated the mechanosensitivity of BCR^Ibsp/Acp5^ mice using an *in vivo* tail-loading model (Fig. 4A). To explore how bone adapts to mechanical cues, we employed weekly time-lapsed micro-CT images complemented by micro-FE analysis. This approach sheds light on tissue-level responses and provides insights into the activities of osteoblasts and osteoclasts despite the observed morphological deformities in the trabecula and the skeleton of BCR^Ibsp/Acp5^ mice.

Fig. 4B displays the representative micro-CT images illustrating the disparity between the last (week 4) and the initial (week 0) time points of loading and reveals the thickening of the trabecula and cortex in both BCR and WT mice in contrast to their respective controls. Time-lapsed micro-CT images indicated a similar increase in static parameters in loaded BCR^Ibsp/Acp5^ and WT mice compared to their sham-loaded (control) counterparts (Fig. 4B and C, Supplementary Table 5). In response to four weeks of loading, trabecular bone volume fraction (BV/TV) exhibited a remarkable increase of +22 % in loaded BCR^Ibsp/Acp5^ mice and +12 % in loaded WT mice compared to their respective controls (Fig. 4B). This change in BV/TV was accompanied by a significant increase in trabecular thickness (+10 % in BCR^Ibsp/Acp5^, +3 % in WT mice), as well as cortical thickness (+6 % in BCR^Ibsp/Acp5^, +5 % in WT mice), and cortical bone area fraction (+6 % in both BCR^Ibsp/Acp5^ and WT mice) (Fig. 4B and C). Furthermore, the difference in trabecular BV/TV between the last and the initial time points accentuated the anabolic effect of mechanical loading within the loaded mice, that was distinctly noticeable through a substantial increase in BV/TV after 4 weeks (+27 % in BCR^Ibsp/Acp5^, +16 % in WT) (Fig. 4D). In contrast, a decrease in BV/TV indicated a catabolic effect of sham-loading (−4% in BCR^Ibsp/Acp5^ and, -3% in WT mice, respectively) (Fig. 4D), which has been reported previously for longitudinal imaging studies including multiple animal handling and anesthesia sessions [28].

Additionally, tissue mineral density (TMD) in both loaded mice increased at the end of 4 weeks of loading compared to their corresponding controls (+0.2 % in BCR^Ibsp/Acp5^ and +0.01 % in WT mice, respectively) (Fig. 4E).

**Fig. 5:**
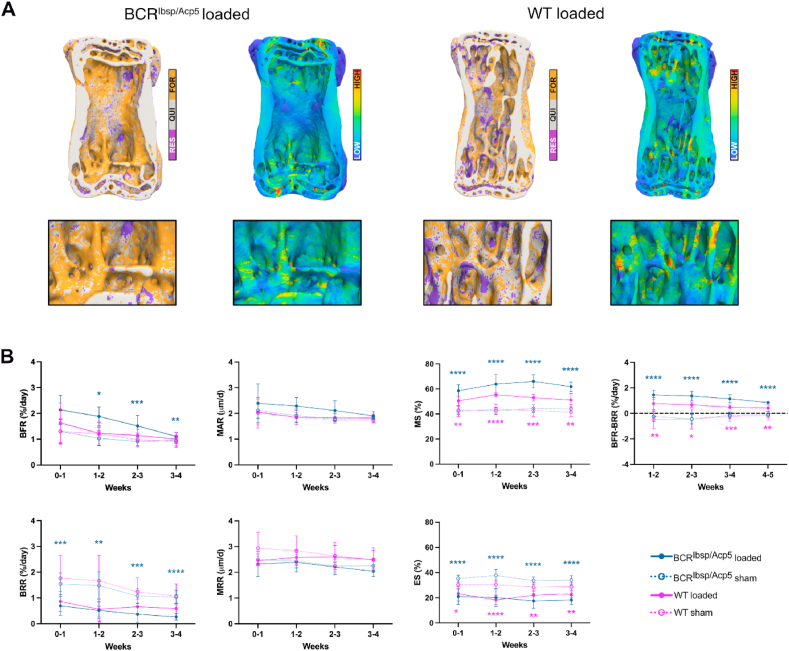
Mechanically driven regulation of the bone remodeling activity of BCR^ibsp/acp5^ mice. A) Visualization of bone remodeling sites (formation, quiescence, and resorption) in the caudal vertebra *in vivo*. Overlay of time-lapsed micro-CT image showing sites of bone formation (orange), quiescence (grey), and resorption (purple) at week 2. The corresponding map of the effective strain highlights the spatial distribution of mechanical strains showing higher and lower (blue) strain regions computed by micro-FE analysis. B) Dynamic bone morphometric parameters in the trabeculae over 4-week tail loading assessed by *in vivo* micro-CT: formation events as BFR, MAR, MS; resorption events as BRR, MRR, and ES. Changes in the net remodeling rate were shown as the difference between BFR and BRR over the 4-week loading period. Data represent mean ± sd (n = 9–10 female mice/group), *< 0.05, **< 0.01, ***< 0.001 according to two-way ANOVA with Dunnett multiple comparison test. (For interpretation of the references to colour in this figure legend, the reader is referred to the Web version of this article.)

Considering the dynamic parameters that quantify the bone formation and resorption activities from time-lapsed micro-CT images, Fig. 5A illustrates a representative visualization of a caudal vertebra indicating bone remodeling sites (formation, resorption, and quiescence) and their corresponding local mechanical signals, shown as effective strain [29]. Based on the analysis of parameters linked to bone formation events, bone formation rate (BFR) was notably higher in loaded BCR^Ibsp/Acp5^ and WT mice compared to their sham-loaded counterparts (+34 % in BCR^Ibsp/Acp5^ and +11 % in WT mice) (Fig. 5B). Moreover, mineral apposition rate [30], that indicates the thickness of formation packages, was not notably different between loaded and sham-loaded groups for both BCR^Ibsp/Acp5^ and WT mice (+11 % in BCR^Ibsp/Acp5^ and +3 % in WT mice, respectively). In contrast, the mineralizing surface [31], that refers to the area of formation sites, was significantly higher in both loaded groups (+30 % in BCR^Ibsp/Acp5^ and +19 % in WT mice) (Fig. 5B). Focusing on the analysis of bone resorption events, bone resorption rate (BRR) exhibited a pronounced increase in sham-loaded groups compared to that in loaded mice (+65 % in BCR^Ibsp/Acp5^ and +52 % in WT mice). Mineral resorption rate (MRR), indicating the depth of resorption cavities, exhibited no significant differences between loaded and sham-loaded mice for both groups (−5% in BCR^Ibsp/Acp5^ and -4% in WT mice). The surface area of eroded surfaces (ES) decreased considerably with loading for the loaded groups (−47 % in BCR^Ibsp/Acp5^ and -40 % in WT mice) (Fig. 5B). Moreover, the net remodeling rate that signifies the difference between BFR and BRR demonstrated that loaded mice exhibited a constant positive remodeling trend throughout all weeks (+1.2 % per day in BCR^Ibsp/Acp5^ and +0.6 % per day in WT mice), thus indicating a bone gain. In contrast, sham-loaded mice exhibited a negative remodeling (−0.2 % per day in BCR^Ibsp/Acp5^ and -0.3 % in WT), that was associated with bone loss (Fig. 5B). For simplicity reasons, we are assuming that both remodeling and modeling events, also referred to as (re)modeling [8,9], contribute to the measurement of the net remodeling rate.

In order to gain a deeper understanding of the cellular-level mechano-molecular mechanism, we also examined the expression of cellular mechanotransduction markers. As YAP-1 and TAZ-Hippo pathway effectors have been demonstrated to function as mechanotransducers where they modulate mechano-responsive gene transcription programs that govern cellular functions [32,33], we assessed TAZ expression in response to mechanical loading on caudal vertebrae. Hoechst staining highlighted the vertebral architecture of loaded and sham-loaded BCR^Ibsp/Acp5^ and WT sections (Fig. S4A). Immunostaining for TAZ exhibited significantly higher expression in loaded groups around the trabeculae than in the sham-loaded controls, particularly in BCR^Ibsp/Acp5^ mice (Figs. S4B–C). Moreover, the serum CTX protein levels from loaded and sham-loaded BCR and WT mice indicated normal levels and were not significantly different between the groups despite the low CTX levels observed *in vitro* (Fig. S4D).

### 2.4 Bone adaptation in BCR^Ibsp/Acp5^ mice is also controlled by the local mechanical *in vivo* environment

**Fig. 6:**
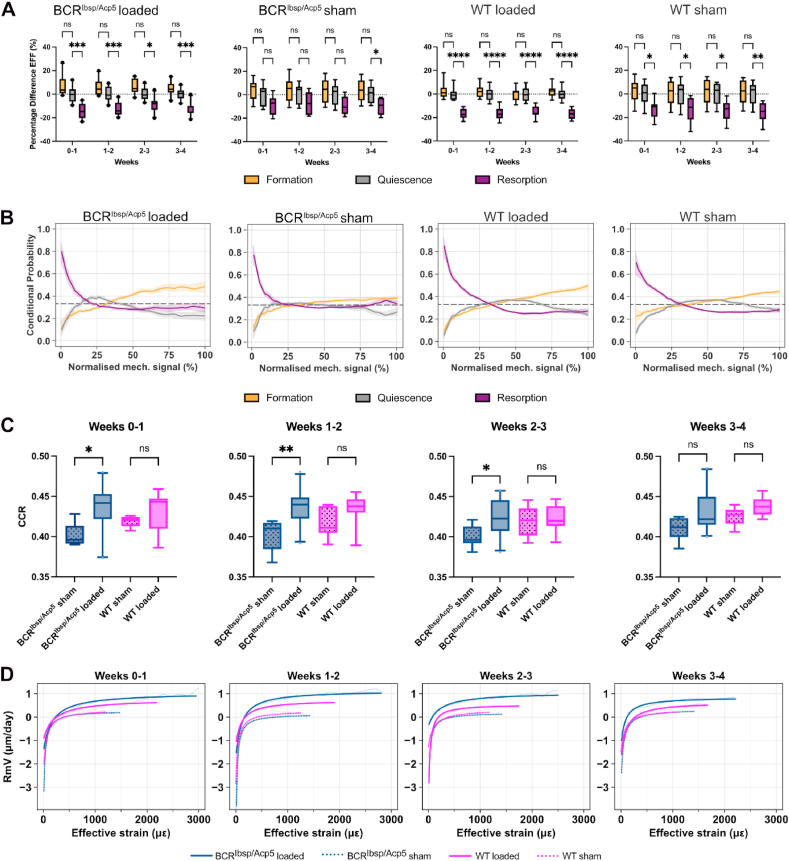
Quantification of mechanoregulation information from time-lapsed *in vivo* micro-CT images of the 6th caudal vertebrae. A) Mean effective strain at formation, quiescent, and resorption sites expressed as the percentage difference from the mean quiescent effective strain for all weekly time points. *< 0.05, **< 0.01, ***< 0.001 according to two-tailed student t-test. B) Conditional probability curves connecting the mechanical environment expressed as normalized effective strain with remodeling events, computed for all groups using micro-FE with homogeneous material properties. The plots show the mean probability line per group after applying a LOWESS operation for the interval 0–4 weeks and its corresponding 95 % confidence interval. The dashed line at 0.33 identifies the probability of a random event for a ternary classification case. C) Comparison of correct classification rate values obtained from conditional probability curves for 0–4 weeks. Higher CCR values indicate a higher sensitivity to retrieve mechanoregulation information. *< 0.05, **p< 0.01, ***< 0.001 according to two-tailed student t-test. D) Estimated mechanostat remodeling velocity curves fitted with continuous hyperbola functions for weekly time points and all groups. Data points are filtered such that at least three mice are averaged per group for each mechanical signal value. Effective strain values are also obtained from micro-FE analysis using homogeneous material properties. (n = 9–10 female mice/group).

Finally, to investigate bone mechanoregulation in BCR^Ibsp/Acp5^ mice, the association between remodeling events and mechanical *in vivo* environment was analyzed. Bone resorption sites were associated with lower effective strain values compared to quiescent clusters in all groups and loading regimens, except for sham-loaded BCR^Ibsp/Acp5^ mice at weeks 3–4 (Fig. 6A). For each remodeling event, the effective strain values for loaded groups exhibited lower variability, as indicated by narrower interquartile ranges in comparison to the sham-loaded groups, thus highlighting a distinct response to mechanical loading and association with remodeling events (Fig. 6A). Similar results were observed in the conditional probability curves that were associated with the likelihood of observing each remodeling event for a given mechanical signal value. Between weeks 0–4, resorption events were strongly associated with lower normalized effective strains (below 25 % for BCR^Ibsp/Acp5^ and below 27 % for WT mice), whereas a higher probability of formation events was concurrent with higher mechanical signal values (Fig. 6B). The loaded groups exhibited a clear separation between formation and quiescence events for higher normalized effective strains (greater than 60 %), and there was a narrower separation between remodeling events in the same interval for sham-loaded groups, particularly for the BCR^Ibsp/Acp5^ mice, where the curves were close to the random probability of 0.33. For all groups and time points, the correct classification rate [26,34] values determined from conditional probability curves were above 0.33, supporting a non-random association between the magnitude of effective strain and remodeling events. Additionally, there were significant differences between loaded and sham-loaded BCR^Ibsp/Acp5^ mice up to week 3 (Fig. 6C), and this was consistent with the strong anabolic response presented in Fig. 5B. Although the loaded WT mice had greater CCR values compared to their sham-loaded counterparts, the differences were not statistically significant.

Furthermore, we assessed mechanoregulation using remodeling velocity (RmV) curves, that associated the average remodeling surface velocity with effective strain. RmV curves obtained for each week resembled the mechanostat function proposed by Frost [5], and they were accurately fitted with hyperbolic functions with normalized root mean squared errors (NRMSE) below 5 % (Supplementary Table 6). From these curves, biologically meaningful remodeling parameters were derived and included the formation and resorption saturation levels (FSL and RSL) that determine the magnitude of these events. Additionally, the remodeling threshold (RmT) and velocity modulus (RmVM) provided a quantitative measure of the mechanosensitivity of the model to the applied loading. The loaded BCR^Ibsp/Acp5^ mice exhibited the highest FSL values across all weeks (Fig. 6D) that were significantly different from those observed in other groups (Supplementary Table 7). Similarly, loaded WT mice exhibited significant differences in FSL compared to that of both sham-loaded groups. RSL values were consistently higher for both loaded groups up to week 2 compared to their sham-loaded counterparts, given the increasing number of formation events for this loading condition that shifted the range of RmV curves towards positive values. Significant differences in RSL were observed between the sham-loaded and loaded groups of BCR^Ibsp/Acp5^ mice during weeks 1–2 and 2–3. However, when comparing the loaded groups of BCR^Ibsp/Acp5^ and WT mice, the differences in RSL were significant only during weeks 2–3 (Supplementary Table 7).

Focusing on mechanosensitivity descriptors, RmVM values were consistently higher in the loaded groups than they were in the sham-loaded groups. As expected, cyclic loading created a closely regulated mechanically driven environment, which emphasizes the dependency of RmV values on the magnitude of local effective strains (Supplementary Table 7). Indeed, there were significant differences between loaded and sham-loaded groups for both BCR^Ibsp/Acp5^ and WT mice up to weeks 2 and 3, respectively, in contrast to the comparisons between loaded or sham-loaded WT and BCR^Ibsp/Acp5^ mice. In weeks 3–4, the loaded groups had adapted to the supraphysiological loading condition, as supported by an increase over time in the Pearson correlation coefficient (PCC) between RmV curves of loaded and sham-loaded BCR^Ibsp/Acp5^ and WT mice. In particular, these values increased from 0.895 to 0.949 (p < 0.0001) for weeks 0–1 to 0.966 and 0.978 (p < 0.0001) at weeks 3–4, for BCR^Ibsp/Acp5^ and WT mice, respectively, supporting comparable bone remodeling responses between these groups except for differences in RmT. Comparably, RmV curves between loaded groups also evolved towards a more uniform bone remodeling response based on the increase in the PCC between the loaded BCR^Ibsp/Acp5^ and WT mice from 0.910 at weeks 0–1 to 0.955 at weeks 3–4. Regarding the RmT parameter, the sham-loaded groups showed consistently higher values in comparison to loaded groups. This indicates that in a mechanically driven environment, there is a precise regulation on the threshold to initiate formation, which restricts resorption events to a narrower interval of effective strain values. BCR^Ibsp/Acp5^ loaded, and sham-loaded mice exhibited significant differences for all weeks, whereas discrepancies between WT mice were significant only at weeks 1–2 and 2–3. Importantly, there were no significant differences observed between BCR^Ibsp/Acp5^ and WT mice in either the sham-loaded or loaded groups at any time point, except for the comparison between loaded groups at weeks 3–4. The similarities in the responses observed in conditional probability curves and between parameters obtained from remodeling velocity curves from BCR^Ibsp/Acp5^ and WT mice reinforce comparable mechanoregulation between these mice.

## 3 Discussion

Numerous studies have provided evidence for the involvement of TRAP in essential molecular processes, including cell recruitment [35], cell attachment [17], and the development of long bones [35,36]. However, the role of TRAP in multiscale bone mechanoregulation remained incompletely understood. Here, we demonstrated that TRAP is not a critical regulator of mechanoregulated trabecular bone remodeling and adaptation. Our findings demonstrated the feasibility of employing CRISPR-Cas9 technology to generate fluorescent reporter (BCR^Ibsp/Acp5^) mice with TRAP deficiency with fluorescent osteoclasts and osteoblasts, thus allowing the identification, tracking, and characterization of tagged cells *in vivo* and *in vitro*. However, despite the absence of TRAP activity unchanged expression of other critical osteoclast-associated genes enabled functional trabecular bone remodeling in a controlled mechanically driven environment in BCR^Ibsp/Acp5^ mice.

TRAP expression in our mice is not expected to be limited to osteoclasts but can also be found in macrophages and other immune cell types. However, the observed phenotype of BCR^Ibsp/Acp5^ mice from our study including trabecular bone impairments and reduced resorption capacity *in vitro* were still remarkably similar to those observed in TRAP knock-out mice [20,[37], [38], [39], [40]]. Moreover, TRAP deficiency in our mice appeared to compromise the trabecular bone structure without altering mineral density, which was inconsistent with the findings from the previous TRAP knock-out mice studies. These studies have suggested that the deficiency in acid phosphatase activity in TRAP knock-out mice impacts the regulation of osteoclast recruitment to the mineralization front through the partial dephosphorylation of osteopontin (OPN) and bone sialoprotein (BSP), thereby potentially resulting in increased mineralization [20]. The difference observed in our mouse model could potentially be attributed to the underestimation of tissue mineral density values acquired through micro-CT because TMD is an average value computed over the entire image which diminishes its specificity.

In addition to providing insights into the skeletal morphology of our TRAP-deficient mice, it is important to emphasize the characterization of fluorescent osteoclasts with TRAP deficiency. Notably, distinct disparities were observed between osteoclasts from BCR^Ibsp/Acp5^ and WT mice; osteoclasts with TRAP-deficiency exhibited smaller and irregular shapes and were more abundant than the osteoclasts from WT mice. Additionally, BCR^Ibsp/Acp5^ mice also exhibited a lower number of nuclei in their osteoclasts than that of osteoclasts from WT mice. However, there was no observable difference in the number of pre-osteoclasts containing two nuclei between the groups, thus indicating that the formation of large, highly nucleated osteoclasts was compromised whereas the formation of pre-osteoclasts remained unaffected in BCR^Ibsp/Acp5^ mice. Additionally, these discrepancies observed in the osteoclasts of BCR^Ibsp/Acp5^ mice may indicate that the fusion process of osteoclasts or their maturation does not proceed as expected, and this likely contributes to the observed clustering of smaller osteoclasts. Furthermore, this potentially disrupted fusion process strongly reduced CTX levels *in vitro*, thus implying a decreased capacity for bone resorption. However, unchanged serum CTX levels in our TRAP-deficient mice, combined with the outcomes from the *in vivo* loading indicated that the bone resorption capacity remained unaffected.

Despite the downregulation of TRAP expression, other key genes involved in osteoclastogenesis have unchanged expression levels (*Calcr, Ctsk, Mmp9*) in both TRAP-deficient and WT osteoclasts. Expression levels of *Calcr, Ctsk, and Mmp9, being* essential for bone resorption and remodeling, displayed only slight differences between the osteoclasts of BCR^Ibsp/Acp5^ and WT mice. Therefore, these small distinctions may contribute to maintaining the stability of osteoclast function for bone resorption in BCR^Ibsp/Acp5^ mice but might not trigger significant alterations in overall osteoclast function and the associated bone phenotype for TRAP deficiency.

Furthermore, we showed that TRAP-deficient BCR^Ibsp/Acp5^ mice exhibited robust mechanosensitivity during caudal vertebra loading, on par with WT mice. Structural and dynamic bone morphometric parameters resulted in equivalent outcomes for both mice, demonstrating that loading had an anabolic effect on bone, as previously observed [22,41,42]. The trabecular bone deformity observed in BCR^Ibsp/Acp5^ mice, along with their lower trabecular BV/TV could explain their stronger response to mechanical loading. Despite receiving the same mechanical dose as WT mice, BCR^Ibsp/Acp5^ mice, which had initially lower trabecular BV/TV and reduced trabecular structure compared to WT mice, exhibited an increased area of high local surface strains. These high local surface strains are known to be associated with an increased likelihood of bone formation events, thereby may contribute to their increased response to mechanical loading, leading to increased bone formation in response to mechanical stimuli. The observed increase in BV/TV is in agreement with prior findings, where loads of 4 N resulted in substantial BV/TV increases compared to sham and 2 N [43]. Notably, the reported increase with an 8 N load was significantly greater. This suggests that the 8 N load in BCR mice is likely to be deemed supraphysiological, surpassing the threshold for pronounced anabolic responses, even within the initially lower trabecular BV/TV. Additionally, *in silico* bone loading estimations based on micro-FE have determined an axial compressive force of about 4 N as the equivalent loading acting on sham-loaded mice during a loading experiment [44]. This value can be deemed as an upper bound for physiological loading, supporting our assumption that a force of 8 N can be considered supraphysiological. Moreover, the quantification of bone formation and resorption sites also indicated that cyclic mechanical loading mainly influenced the surface area of these events. Therefore, an increase in the area of mineralized surfaces and a reduction in eroded surfaces may also suggest an increased osteoblast activity in BCR^Ibsp/Acp5^ mice. This notion aligns with previous findings where TRAP knock-out mice displayed signs of premature maturation of osteoblasts [36] and higher mineralization of bone matrix due to the partial dephosphorylation of OPN and BSP mentioned earlier [17]. Thus, downregulation of TRAP in osteoclasts from BCR^Ibsp/Acp5^ mice could increase anabolic responses due to increased activity of osteoblasts.

We further provided evidence for the comparable expression of mechano-molecular markers at the cellular level. TAZ expression increased in loaded WT and BCR^Ibsp/Acp5^ mice, more prominently in BCR^Ibsp/Acp5^ mice, thus indicating a robust reaction of TRAP-deficient mice to cellular mechanical cues. Upon osteoblast differentiation, TAZ activity was increased, ultimately leading to its interaction with RUNX2 and subsequent activation of the *Runx2* transcription pathway, thereby playing a significant role in the regulation of osteoblastogenesis [45,46]. In our study, increased TAZ expression in loaded BCR^Ibsp/Acp5^ mice may be associated with increased osteoblast activity due to TRAP deficiency in response to mechanical stimuli.

Consistent with these results, we also reported comparable mechanoregulation in BCR^Ibsp/Acp5^ and WT mice. Previous studies have applied mechanoregulation analysis to evaluate the effects of cyclic mechanical loading in the vertebrae [8,9,47], femur [26,48], and tibia [23,49] of WT (C57Bl/6J) mice. Similarly, this analysis highlighted a similar EFF distribution of remodeling clusters between our TRAP-deficient and WT mice, where the mean EFF at formation sites was higher than that at quiescent sites and lower than that at resorption sites in both the loaded and sham groups. In both loading groups, these differences were significant, thus suggesting that mechanical cues strongly influenced the remodeling. As the CCR values, remodeling velocity curves, and derived parameters were indicated to capture distinct responses to cyclic mechanical loading at varying loading frequencies, we anticipate that this analysis is sensitive to differences in mechanically driven bone adaptation between BCR^Ibsp/Acp5^ and WT mice. Notably, lower RmVM values for sham groups described a flatter RmV response, aligning with the observations from conditional probability curves where formation and quiescent events were closer to the random probability for higher normalized effective strains and suggesting an increased number of non-targeted remodeling events that are rather stochastic than mechanically-driven [50,51]. Concurrently, the increase in formation events in response to supraphysiological loading shifted RmV curves towards higher velocity values and was followed by lower RmT values. As RmV curves describe bone remodeling events at the micrometer scale, RmT values mark the transition between low strains where resorption is primarily observed and high strains where the formation, on average, occurs more often and to a greater extent. Importantly, the trends observed in our data align with the original predictions by Frost [5], who argued that increased mechanical demands could decrease the magnitude of this parameter. Frost further argued that the region around RmT would define an adapted window [5], also described as a lazy zone [49], that is a range of strains where bone formation and resorption would balance each other. In the mouse bone and with the resolution of the micro-CT used here, non-targeted remodeling and mechanically driven modeling events are analyzed collectively. Such interval, if existent, would appear as a uniform RmV region bounded by monotonic changes in RmV for lower and higher effective strains. In agreement with previous results [8,23,25], our findings provided no evidence of a lazy zone for any group or loading condition.

Conversely, mechanical unloading, often performed through hindlimb suspension or immobilization, leads to loss of bone mass and strength [52]. This loss of bone induced by unloading stems from the decoupling of bone turnover, where bone formation diminishes, while bone resorption increases [53]. In BCR^Ibsp/Acp5^ mice, already afflicted with TRAP deficiency impairing their osteoclast function, unloading could lead to significant bone loss. TRAP plays a vital role in osteoclast function, and its absence in BCR^Ibsp/Acp5^ mice potentially renders them susceptible to bone loss during unloading.

Exercise is well-known to exert a positive influence on bone health [54]. Weight-bearing activities and resistance training have been scientifically demonstrated to stimulate bone remodeling and enhance bone density [54]. In the context of BCR^Ibsp/Acp5^ mice, their response to exercise would likely resemble the results observed after *in vivo* mechanical loading, where they exhibit increased bone formation similar to WT mice. This connection is based on the findings that exercise, particularly resistance training such as treadmill running, has been shown to increase bone formation and tissue-level mechanical properties in young mice [54]. Despite the TRAP deficiency that affects the osteoclast function in BCR^Ibsp/Acp5^ mice, the beneficial effects of exercise on bone formation may persist. Consequently, exercise could potentially play a role in maintaining or even improving bone mass in our TRAP-deficient mice, and thus aligning their response with that of WT mice in terms of increased bone formation.

Hence, our findings provide novel insights into bone mechanoregulation and emphasize that TRAP is not the primary regulator of this process. Importantly, we observed that TRAP deficiency in BCR^Ibsp/Acp5^ mice did not alter the functional remodeling activities in the trabecular region. Our results shed light on the mechanomolecular mechanism of TRAP and demonstrated that TRAP-deficient mice exhibit a similar response to mechanical loading and expression of molecular markers involved in bone mechanosensitivity. Additionally, our study highlighted the feasibility of simultaneous identification and tracking of fluorescently labeled osteoblasts and TRAP-deficient osteoclasts *in vivo* and *in vitro* using CRISPR/Cas9 mediated fluorescent reporter (BCR^Ibsp/Acp5^) mice. The ability of TRAP-deficient osteoclasts to sustain bone remodeling in a mechanically driven environment suggests that TRAP may not be an exclusive target for therapeutic strategies aimed at bone diseases. Future pharmacological interventions could explore the modulation of associated pathways rather than direct TRAP inhibition. The observed bone remodeling and mechanosensitivity that occurred despite TRAP deficiency indicate that other genetic or environmental factors may compensate for the lack of TRAP. Therefore, it is important to focus on exploring these factors and investigating the osteoclast physiology, development, and function as novel therapeutic targets to restore the altered phenotype and activity of osteoclasts.

## 4 Limitations of the study

In this study, the presence of the fusion of the TRAP + mCherry complex disrupted the TRAP function and hindered our ability to fluorescently label osteoclasts with functional TRAP expression. Although *in silico* analysis suggested an absence of off-target effects, this does not entirely preclude their presence *in vivo* which could potentially confound our findings. In addition, this study focused primarily on the trabecular bone and did not examine the effects of TRAP deficiency on cortical bone in detail. Additionally, RNA sequencing results could be validated by performing qPCR to confirm the differential regulation of osteoclast target genes and to quantify the TRAP expression levels. The sample size used in the differential gene expression analysis may have affected the statistical power to detect the subtle differences in gene expression among the strains. However, small sample sizes are common in *in vitro* studies involving primary cells; however, studies utilizing larger sample sizes are required to validate these findings. Further investigations are warranted for an in-depth analysis of the interaction between osteoblasts and TRAP-deficient osteoclasts. This will provide a more comprehensive understanding of the cellular mechanisms involved and their impact on bone remodeling and mechanosensitivity. Furthermore, we still observed an anabolic response in loaded versus control indicating that loading was effective in these mice. While it could be argued that the exact force may be higher than the same force used on WT mice with bigger vertebrae, our goal was to evaluate whether loading would still have an effect on bone remodeling, rather than determining the minimum load capable of producing anabolic effects. Regarding the *in silico* analysis, the error in identifying remodeling volumes and surfaces from 3D time-lapsed micro-CT images was observed to be approximately 5 % and 18 %, respectively, and this may influence the mechanoregulation analysis performed based on conditional probability and remodeling velocity curves. Additionally, micro-FE simulations only consider a force applied along the longitudinal axis of the vertebra and femur, and this emulates the *in vivo* loading scenario but neglects possible contributions from bending or shear components.

## Star ★ methods

•KEY RESOURCES TABLE
•RESOURCES AVAILABILITY
  oLead contact
  oMaterials availability
  oData and code availability
•EXPERIMENTAL MODELS AND SUBJECT DETAILS
  oAnimal Models
  oEthics Statement
•METHODS DETAILS
  oBone sample preparation and immunofluorescence staining
  oImmunohistochemistry for bone samples
  o*In vitro* osteoclast differentiation
  oRNA sequencing and data analysis
  oAnalysis of bone resorption marker
  oCyclic mechanical loading of the sixth caudal vertebra
  oMicro-computed tomography (micro-CT) analysis
  oAutomated compartmental analysis of the caudal vertebrae
  oMicro finite element (micro-FE) analysis
  oMechanoregulation analysis
•QUANTIFICATION AND STATISTICAL ANALYSIS

## STAR ★ METHODS

**Table:** Key Resource Table

REAGENT OR RESOURCE	SOURCE	IDENTIFIER
Antibodies		
Rabbit mCherry	Invitrogen, Thermo Fischer Scientific	Cat#:PA5-34974
Rabbit GFP	Abcam	Cat#: ab290
Rabbit IBSP	Abcam	Cat#: 270457
Rabbit TAZ	Sigma-Aldrich	Cat#: HPA007415
Anti-Rabbit Alexa Fluor 647	Abcam	Cat#: ab150075
Anti-Rabbit IgG Biotinylated	Abcam	Cat#: ab6720
Phalloidin Alexa Fluor 555	Thermo Fischer Scientific	Cat#: A30106
Phalloidin Cruz Fluor 647 Conjugate	Santa Cruz	Cat#: sc-363797
Chemicals, peptides, and recombinant proteins		
Rabbit IgG Isotype	ThermoFischer Scientific	Cat#: 02-6102
Normal donkey serum	Abcam	Cat#: ab7475
Hoechst 33342	Sigma-Aldrich	Cat#: B22126-25G
Calcein Blue	Sigma-Aldrich	Cat#: M1255
Fast Green	Sigma-Aldrich	Cat#: F2758
Toluidine Blue	Roth	Cat#: 0300.1
Prolong Diamond	Thermo Fischer Scientific	Cat#: P36961
DPX Mounting Medium	Sigma-Aldrich	Cat#: 44581
EDTA	Sigma-Aldrich	Cat#: E5134
Sucrose	Sigma-Aldrich	Cat#: S7903
Polyvinylpyrrolidone (PVP)	Sigma Aldrich	Cat#: P5288
16 % Formaldehyde (methanol free)	Thermo Fischer Scientific	Cat#: 28908
PBS, ph:7.4	GIBCO, Thermo Fischer Scientific	Cat#: 10010-015
Xylene	Sigma-Aldrich	Cat#: 534068-4L
Paraformaldehyde	Thermo Fischer Scientific	Cat#:J19943.K2
Neutral Buffer Formalin	Sigma-Aldrich	Cat#: HT501128
Tween 20	Sigma-Aldrich	Cat#: P1379
Peroxidase	Thermo Fischer Scientific	Cat#: 855910
Bovine Serum Albumin heat shock fraction, pH 7, ≥98 %	Sigma-Aldrich	Cat#: 7906-10G
Alpha-MEM	GIBCO, Thermo Fischer Scientific	Cat#: A10490
VECTASTAIN Elite ABC-HRP Kit, Peroxidase	Vector Laboratories	Cat#: PK-6100
Metal-enhanced DAB Substrate Kit	Thermo Fischer Scientific	Cat#: 34065
GlutaMAX Supplement	GIBCO, Thermo Fischer Scientific	Cat#: 35050061
Fetal Bovine Serum	GIBCO, Thermo Fischer Scientific	Cat#: 10270-106
Glass-bottom 96 well plate	Greiner	Cat#: 655090
Penicillin Streptomycin Fungizone (Amphotericin B), Antibiotic-Antimycotic	GIBCO, Thermo Fischer Scientific	Cat#: 15240062
Recombinant Mouse Rankl	RD Systems	Cat#: 462-TEC
Recombinant Mouse MCSF	RD Systems	Cat#: 416-ML
Mouse CTX-I ELISA kit	Immunodiagnostic System Ltd	Cat#: AC-06F1
Critical commercial assays		
TRAP	Sigma-Aldrich	Cat#:387-A
RNeasy Micro Kit	QIAGEN	Cat#:74004
Deposited data		
RNA seq data	This paper	
Experimental models: Cell lines		
Primary mouse osteoclasts	This paper	N/A
Experimental models: (Organisms/strains)		
C57BL6/J	Janvier Labs	N/A
BCR^Ibsp/Acp5^*(C5*7BL*/6j.Ibsp*^*em1(ETHZ)*^*.Acp5*^*em1(ETHZ)*^*)*	This paper	N/A
BCR^Ibsp/Acp5^*(C5*7BL*/6j.Ibsp*^*em1(ETHZ*^*)*	This paper	N/A
Software and algorithms		
GraphPad Prism 9	GraphPad Software	N/A
Affinity Designer	Affinity Software	N/A
Fiji	ImageJ Software	N/A
IPL (Image Processing Language)	Scanco Medical AG	N/A

## Resource availability

### Lead contact

Requesting further information and resources in this study should be addressed to ram@ethz.ch.

### Materials availability

The mice in this study can be requested from the lead contact.

### Data availability

The RNA-sequencing data were deposited to ArrayExpress under the accession number E-MTAB-11403 [55].

### Code availability

The code is available upon reasonable request from the lead contact.

## Experimental model and subject details

### Ethics statement

All animal procedures were approved by the local authorities (animal license number ZH09/2018, Verterinäramt des Kantons Zürich, Zurich, Switzerland) and comply with the ARRIVE guidelines. All mice were maintained for animal husbandry, welfare, and monitoring at the ETH Phenomics Center (12:12 h light-dark cycle, maintenance feed-vitamin fortified pelleted diet (3437 KLIBA NAFAG Kaiseraugst, Switzerland) and water ad libitum, three to five animals per cage).

### Animal models

Bone Cell Reporter (BCR) mouse lines used in this study were initially designed as knock-in models with the aim of labeling osteoclasts (*Acp5*, mCherry) and osteoblasts (*Ibsp*, eGFP) respectively. BCR^Ibsp/Acp5^ (*C5*7BL*/6j.Ibsp*^*em1(ETHZ)*^*.Acp5*^*em1(ETHZ)*^) and BCR^Ibsp/WT^ (*C5*7BL*/6j.Ibsp*^*em1(ETHZ*^) mice were derived from four founder animals generated via CRISPR/Cas9 genome editing (Figs. S5A–C). Tartrate-resistant acid phosphatase type 5 - *Acp5* and integrin-binding sialoprotein - *Ibsp* were labeled with fluorescent proteins (mCherry, eGFP), respectively. Donor templates were designed to contain a furin recognition site (RAKR), a self-cleaving peptide (P2A), and the fluorescent reporter sequence (eGFP/mCherry) flanked by homology arms of 1000bp, which were specific to sequences upstream (5′) and downstream (3′) of the target gene's stop codon (ensembl.org; Gene IDs, GenBank: 11433 for Acp5, 15891 for Ibsp). and inserted into puC57 vectors (ShineGene Molecular Biotech, Inc., Shanghai, China). Guide RNAs were selected based on specificity, efficiency, off-target predictions, and distance from the insertion site, using open software (http://crispor.tefor.net, https://chopchop.cbu.uib.no) [57]. After confirming the functionality of guide RNAs (Acp5: GCAGGACTCTCGTGGTGTTCAGG and Ibsp: CGGGGAGGGGGCTTCACTGATGG) and Cas9 protein through *in vitro* digestions, CRISPR/Cas9 reagents were injected into C57BL/6J zygotes (Zygotes Kit, Janvier Labs, Le Genest-Saint-Isle, France) and transferred to Swiss Webster recipient mice. Genotyping was performed for the mouse litters using PCR followed by Sanger sequencing (Microsynth, Balgach, Switzerland). Heterozygous F1 animals were crossbred to obtain homozygous animals, and the BCR mouse lines were maintained in homozygosity. Homozygous offspring from the BCR^Ibsp/Acp5^ line exhibited an intact TRAP-mCherry complex; thus, the BCR^Ibsp/Acp5^ mice displayed TRAP knock-down-like characteristics at the protein level, with osteoclasts lacking TRAP expression. Despite this, both osteoblasts and osteoclasts continued to express the fluorescent proteins (eGFP and mCherry). All BCR mouse lines included in this study were genotyped prior to experiments (Transnetyx, Cordova, USA). Age-matched C57BL/6J male and female mice purchased from Janvier Labs were used as controls (WT) for all experiments [56].

### Bone sample preparation and immunofluorescence staining

Mice were euthanized and femurs and vertebrae were harvested from 5- and 20-week-old mice and fixed immediately in ice-cold 4 % paraformaldehyde (PFA) for 24 h at 4 °C. Bones were decalcified in 12.5 % EDTA for 10–11 days at 4 °C, followed by overnight incubation in sucrose solution (20 % sucrose, 2 % PVP) and embedded in OCT. Samples were stored at −80 °C until sectioning. 10-50 μm-thick cryosections were obtained for immunofluorescence staining. Bone sections were hydrated with PBS 3 times in 5 min intervals and permeabilized with 0.3 % Triton X-100 in PBS for 20 min at room temperature (RT). Slides were incubated in blocking solution (5 % specific serum in 0.025 % Triton X-100) for 45 min at RT, followed by incubation of primary antibody diluted in blocking solution (see Key resources table) overnight at 4 °C. The next day, slides were washed in PBS 3 times in 10 min intervals, followed by incubation in species-specific Alexa Fluor conjugated secondary antibodies diluted in 0.3 % BSA in PBS (together with Hoechst) for 75 min at RT. Slides were incubated in phalloidin-conjugated Alexa Fluor 555 and Hoechst in 0.3 % BSA in PBS for 1 h to stain the actin cytoskeleton. Slides were washed in PBS 3 times in 10 min intervals, air-dried, mounted with Prolong Diamond mounting medium, and the edges were sealed with nail polish.

### Immunohistochemistry for bone samples

Femurs and vertebrae were fixed in 10 % NBF for 24 h, decalcified with 12.5 % EDTA for 10 days, and embedded with paraffin. 5 μm thick paraffin sections were prepared for stainings. Slides were deparaffinized in Xylene 3 times with 5 min intervals, rehydrated in the gradient of EtOH (100 %–70 % with 5 min intervals each), and blocked in 10 % goat serum for 1 h at RT. Slides were stained with mCherry antibody (see Key resource table) overnight at 4 °C. After washing with PBST, the corresponding biotinylated secondary antibody was added for 1 h at RT. DAB was used as chromogen, and Fast Green was used as a counterstaining.

To identify osteoclasts, TRAP staining was performed according to the manufacturer's guidelines, and Fast Green was used as a counterstaining for better visualization of bone architecture.

Calcein blue staining for minerals was used to visualize bone architecture. Slides were stained in 30 mg/ml of Calcein blue prepared in 2 % NaHCO_3_ solution for 10 min, followed by rinsing with PBS 3 times at 10 min intervals and applying DPX mountant to the slides.

### *In vitro* osteoclast differentiation

Femurs were collected from 5-week-old male mice and, after removing surrounding connective tissue and epiphysis, bone marrows were flushed using culture media (αMEM containing 10 % FBS, 1X GlutaMAX, and 1 % Antibiotic cocktails (Streptomycin and Penicillin)). The harvested marrow cells were centrifuged at 600×*g* for 5 min at RT to pelletize them. The resulting supernatant was discarded, and the cells were rinsed using 4.5 ml of cold ddH_2_O to lyse red blood cells, followed by the addition of 500 μl of 10× PBS to adjust osmolarity. Cells were centrifuged again at 600×*g* for 5 min at RT resuspended in 1 ml of culture media with 10 ng/mL MCSF seeded in a non-adherent plate and incubated for 24 h at 37 °C. The following day, the supernatant is collected which contains the non-adherent hematopoietic progenitors [57,58]. Cells were centrifuged at 600×*g* for 5 min at RT and resuspened in 5 mL medium (αMEM with ascorbic acid, 10 % FBS, 1X GlutaMAX, and 1 % Antibiotic cocktails (Streptomycin and Penicillin) and 30 ng/ml MCSF and seeded for osteoclast differentiation. Cells were counted with Trypan blue and seeded in triplicates as 5000 cells/well in a 96-well plate and supplemented with 4 ng/ml of RANKL and 30 ng/ml of MCSF without RANKL as controls. Media was changed on day 3, and multinucleated osteoclasts appeared on day 4.

### F-actin and TRAP staining

To stain F-actin, BCR^Ibsp/Acp5^ and WT osteoclasts (initially seeded at 5000 cells/well) were fixed on day 4 with 4 % PFA for 5 min, permeabilized with 0.1 % Triton in PBS, blocked with 2 % BSA for 1 h, and co-stained with Phalloidin conjugated with Alexa Fluor647 and Hoechst for 45 min. Cells were washed in 0.1 % Triton in PBS, air-dried, and imaged with Nikon wide-field microscopy. Next, the cells were washed with 1X PBS and stained for TRAP according to the manufacturer's guidelines, and imaged with Nikon wide-field microscopy again.

### Osteoclasts resorption assay

Bone slices (Immunodiagnostic System Ltd) were placed in a 96-well plate a day before the experiments, and cells were seeded on them in triplicates. Cells were cultured on the bone slices following the standard osteoclast differentiation protocol (20.000 cells/well in a 96-well plate and supplemented with 4 ng/ml of RANKL and 30 ng/ml of MCSF to induce osteoclast formation, and without RANKL as controls). On day 9, bone slices were fixed with 4 % PFA for 5 min in RT, and resorption pits were imaged with light microscopy. Bone slices with no cells were used as a control.

### RNA extraction from osteoclasts

Cells seeded as 10.000 cells/well and differentiated into osteoclasts following the standard protocol were used for RNA extraction. The medium was aspirated from the cells when they were at day 4 of differentiation and were washed with 1X PBS twice. Next, cells were harvested with Trizol, and the RNA isolation protocol was performed according to the manufacturer's guidelines (Qiagen Micro kit).

### RNA sequencing and data analysis

RNA quality from the isolated cells was checked using a 2100 BioAnalyzer (Agilent). The TruSeq Stranded Total RNA Library Prep Kit (Illumina), according to the manufacturer's instructions, was used for the preparation of sequencing libraries. The single-end sequencing of samples was carried out at the Functional Genomics Center, Zurich (https://fgcz.ch/). Quality assessment of raw sequence data of all samples was analyzed by FastQC (Version: FastQC v0.11.9, (http://www.bioinformatics.babraham.ac.uk/projects/fastqc/). The raw reads were mapped to the mouse reference genome using STAR aligner (Version = 2.7.10a). The mouse genome GRCm39 was downloaded from Ensembl (https://www.ensembl.org/Musmusculus) and the mouse genome index was created from STAR genomeGenerate command [59]. The aligned reads were quantified per gene basis using HTSeq with the following settings (Version: HTSeq-1.99.2; [htseq-count-mode = intersection-nonemptystranded = reverse]) [60]. The RNA-sequencing data were deposited to ArrayExpress under the accession number E-MTAB-11403 [55].To explore the similarities and dissimilarities between the samples, the quantified count data were normalized using the Variance Stabilizing Transformation (VST) and performed Principal component analysis. Further, differentially expressed genes are listed with FDR-adjusted p-value cutoff <0.1 with different contrast (BCR^Ibsp/Acp5^-M vs WT-M, BCR^Ibsp/Acp5^ vs WT, WT vs WT-M, BCR^Ibsp/Acp5^ vs BCR^Ibsp/Acp5^-M) DESeq2 (BioConductor version3.14The Ensembl ID was annotated to gene symbols and Entrez Gene using biomaRt (BioConductor version 3.14). Lists of differentially regulated genes for the different contrasts are provided in Supplementary Table 3.

Gene-set enrichment analysis was performed using Generally Applicable Gene-set Enrichment (GAGE; BioConductor version 3.14) [61]. For functional annotation, we used gene sets from org.Mm.eg.db, a genome-wide annotation package for mice (Bioconductor, version 3.14) [62]. The analysis was performed based on one-on-one comparisons between BCR^Ibsp/Acp5^ vs WT dataset, where WT was used as a control sample. The significant GO terms were listed using an FDR-adjusted p-value <0.01. Lists of enriched terms are provided in Supplementary Table 4.

### Measurement of CTX from *in vitro* osteoclast cultures

The degradation of C-terminal telopeptides of type I collagen (CTX) in the cell culture supernatant from BCR^Ibsp/Acp5^ and WT osteoclasts was measured using Mouse CTX-I ELISA Kit (Immunodiagnostic System Ltd) according to the manufacturer's guidelines. The results were obtained from 3 biological and 3 technical triplicates per group.

### Measurement of serum CTX

Blood was collected from loaded and sham-loaded BCR^Ibsp/Acp5^ and WT mice by cardiac puncture under anesthesia. Subsequently, the collected blood was allowed to clot for 1 h at RT and the resulting clot was removed by centrifugation at 2000×*g* for 10 min at 4 °C. The serum was promptly collected and stored at −80 °C. The measurement of CTX levels was carried out using the Mouse CTX ELISA kit (Immunodiagnostic System Ltd) following the guidelines provided by the manufacturer.

### Cyclic mechanical loading of the sixth caudal vertebra

The 6th caudal vertebra was subjected to cyclic mechanical loading previously described [22,42]. Stainless steel pins were inserted into the fifth and seventh caudal vertebra of 12-week-old female mice (n = 37). After 3 weeks from pinning, mice received either sham (0 N, n = 18) or 8 N (n = 19) cyclic loading with 10 Hz frequency for 5 min, thrice per week over four weeks.

### Analysis of micro-computed tomography images

For the analysis of *ex vivo* femur and vertebra samples, femurs and vertebrae were fixed overnight in 4 % PFA at 4 °C and placed into 70 % EtOH, scanned using micro-CT (micro-CT 40, Scanco Medical AG) with an isotropic voxel-size of 10 μm and 6 μm, respectively; 55 kVp, 145 μA, 200 ms integration time. A Gaussian filter (support: 1) with sigma of 0.8 and 1.2 was applied to 3D image data, after which images were segmented with a threshold of 392 and 580 mgHA/cm^3^, for femurs and vertebrae, respectively [26]. Relevant cortical and trabecular compartments were identified as previously described [22]. A mask of the outer vertebra is obtained by iterative dilation and erosion operations that smooth and close all holes on the surface. Regarding the trabecular and cortical compartments, a distance transform map is computed on the binary image of the caudal vertebra yielding two masks. The growth plates are discarded by cropping the outer 10 % of the inner mask and 12 % of the outer mask. The middle 33 % of the inner mask is also removed to eliminate this nearly empty space of the trabecular region. Evaluation of standard bone morphometric parameters on these regions and whole bone was performed (Supplementary Tables 1–2).

For the analysis of the 6th caudal vertebrae *in vivo,* mice were anesthetized with isoflurane (induction: 4.5–5%, maintenance: 2–2.5 % isoflurane/oxygen) and were scanned weekly via *in vivo* micro-CT (vivaCT-80, Scanco Medical AG) with a resolution of 10.5 μm (55 kVp, 145 μA, 350 ms integration time) for 4 weeks. Registration of micro-CT images from consecutive time points was performed using the image processing language (IPL Version 5.04c; Scanco Medical AG, Switzerland). Similar to the *ex vivo* analysis, standard and dynamic bone morphometric parameters were evaluated for trabecular, cortical, and whole bone, after identifying the corresponding compartments for each image (Supplementary Table 5). Briefly, bone structural parameters were calculated with a direct 3D model-independent algorithm where spheres are fitted at each voxel to quantify trabecular thickness (Tb.Th), trabecular separation (Tb.Sp), trabecular number (Tb.N). Additionally, the following parameters are measured using the binary bone image: total volume (Tb.TV), bone volume fraction (BV/TV), specific bone surface (BS/BV), structure model index [63], connectivity density (Conn.D) [64], and degree of anisotropy (DA) [65,66]. Dynamic parameters are obtained by overlaying registered images, revealing sites of bone formation, quiescence and resorption between the time points considered. While quiescent bone voxels are present in both images, formation sites are voxels only present in the follow-up image and resorption sites are voxels only present in the baseline image. By analyzing the voxels from each remodeling event separately, dynamic parameters describing the surface area, thickness, and remodeling rates can be computed, namely: mineralization surface [31], mineral apposition rate [30] and bone formation rate (BFR) for the formation and eroded surface (ES), mineral resorption rate (MRR) and bone resorption rate (BRR) for resorption.

### Micro finite element analysis of the caudal vertebrae images

All time points of each sample were analyzed with micro-FE to estimate the mechanical signal in the structure in the form of effective strain [67]. The simulations used a Python pipeline previously developed [47], considering homogeneous material properties for bone [42] (Young's modulus of 14.8 GPa and Poisso's ratio of 0.3). Briefly, image voxels were converted to 8-node hexahedral elements, and bone was assumed to behave within the linear elastic regime. Two cylindrical discs were added at the distal ends of the vertebra model, mimicking the role of the intervertebral discs. The bottom nodes of the FE mesh were constrained in all directions, while the top nodes were displaced by 1 % of the length in the z-axis (longitudinal axis of the sample). Effective strain was determined after linear rescaling to match the forces applied *in vivo*: 4 N (physiological loading estimate) [44] for sham-loaded and 8 N for loaded groups. The simulations ran on the Euler cluster operated by Scientific IT Services at ETH Zurich, using the micro-FE solver ParOSol [68], on Intel Xeon Gold 6150 processors (2.7–3.7 GHz).

### Mechanoregulation analysis in the local *in vivo* environment

The mechanoregulation analysis focused on conditional probability curves and remodeling velocity curves to characterize the association between remodeling events and local mechanical signals. Conditional probabilities were computed as previously described [8], for weekly intervals (e.g., weeks 2–3 considered the micro-CT images from weeks 2 and 3 and the micro-FE from week 2) and for the 4-week observation period (considering the micro-CT images from weeks 0 and 4 to identify remodeling events and using micro-FE data from week 0). Effective strain values were normalized by the 99th percentile of all observed values, defining a normalized mechanical signal (%). A conditional probability of 0.33 indicates an equal probability of any remodeling event occurring. The correct classification rate [26,34] metric summarizes the accuracy of the ternary classification problem that classifies remodeling events (formation, quiescence, and resorption) within the range of observed local mechanical signal values. CCR values above 0.33 indicate that the mechanical signal descriptor considered can predict remodeling events with better accuracy than a random classifier.

Additionally, remodeling velocity curves associating the expected surface velocity based on the mechanical signal were determined, as previously described [47]. Remodeling velocity curves were fitted with a hyperbola function, from which biologically meaningful remodeling parameters can be estimated, namely, formation and resorption saturation levels (FSL and RSL) that bound the magnitude of these events and a remodeling threshold (RmT) and a velocity modulus (RmVM), which quantitatively characterize the mechanosensitivity of the model. The curve fit quality was assessed with the normalized root mean squared error (NRMSE, %), considering the interval between the maximum and minimum remodeling velocities observed as the normalization factor.

### Quantification and statistical analysis

Stained bone sections were imaged with Leica SP8 and ZEISS LSM 880 confocal microscope with 10× and 20× air objectives. The selection of ROIs within the femurs and vertebrae are carefully defined as 25–35 % of the length from the growth plate considering the differences in the growth plate (wider in BCR^Ibsp/Acp5^ mice) and the length of the femurs. For both strains, we normalized the imaged area and volume to the total length of femurs to ensure the ROIs were proportionally comparable. Images were analyzed, quantified, and processed using LASX, Zen Blue, and Affinity Designer software. Images were preprocessed with Fiji, and colocalization analysis was performed as described elsewhere [27,69]. The total fluorescent intensity ratio was determined with Fiji software.

*In vitro* osteoclasts were imaged in a glass-bottom 96 well plate using NIKON Wide-Field microscope with 5× and 10× air objectives; the whole surface of the wells was scanned to generate a tile image. Multinucleated cells containing at least 3 nuclei were identified visually and counted with Fiji using Cell Counter plugin, and processed with Affinity Designer software. GraphPad Prism software was used for statistical analysis. All data are presented as mean ± s.d. unless indicated otherwise. Sample numbers were chosen by power analysis for bone morphometry parameters from previous experiments [9]. Reproducibility was ensured based on several independent experiments. None of the animals were excluded from the analysis. Statistical significance for all datasets was denoted by p < 0.05. The following statistical tests were considered: two-way ANOVA with Dunnett's comparison test for *in vivo* experiments, two-tailed student's t-test for *ex vivo* histology and Mann-Whitney test for *in vitro,* and one-way ANOVA with Dunnett's comparison test for *ex vivo* micro-CT analysis.

Regarding the *in silico* analysis, as previously described [47], a hyperbola function was fitted to the remodeling velocity curves per group and weekly time points, and a balanced bootstrap approach was used to characterize the distribution of the parameters estimated from the curve fits. Group comparisons of these distributions were performed for all weekly time points and group pairs. Due to the skewness of some of the distributions generated, statistical comparisons considered the bias-corrected and accelerated bootstrapped confidence intervals [70]. As indicated above, p < 0.05 was considered significant and was adjusted for multiple comparisons using Bonferroni correction. Additionally, correlations between remodeling velocity curves from different groups were assessed with Pearson Correlation Coefficient (PCC).

Sample sizes were selected based on our previous experiments and a series of independent experiments were carried out to ensure our findings could be consistently replicated.

## CRediT authorship contribution statement

**Dilara Yılmaz:** Writing – review & editing, Writing – original draft, Methodology, Investigation, Formal analysis, Data curation, Conceptualization. **Francisco C. Marques:** Writing – review & editing, Writing – original draft, Methodology, Formal analysis, Conceptualization. **Yannick Fischer:** Formal analysis, Data curation. **Sandra Zimmermann:** Formal analysis, Data curation. **Gaonhae Hwang:** Formal analysis, Data curation. **Penny R. Atkins:** Writing – review & editing, Methodology, Formal analysis. **Neashan Mathavan:** Writing – review & editing, Data curation. **Amit Singh:** Writing – review & editing, Formal analysis, Conceptualization. **Pedro P.C. de Souza:** Writing – review & editing, Formal analysis, Data curation, Conceptualization. **Gisela A. Kuhn:** Supervision, Methodology, Investigation, Data curation, Conceptualization. **Esther Wehrle:** Writing – review & editing, Investigation, Funding acquisition, Conceptualization. **Ralph Müller:** Writing – review & editing, Supervision, Resources, Project administration, Methodology, Funding acquisition, Conceptualization.

## Declaration of AI and AI-assisted technologies in the writing process

During the preparation of this work, the authors used Chat GPT, an AI language model developed by Open AI to guide the improve the readability of this manuscript. After using this tool/service, the author(s) reviewed and edited the content as needed and took full responsibility for the content of the publication.

## Declaration of competing interest

The authors declare the following financial interests/personal relationships which may be considered as potential competing interests: Prof. Ralph Mueller reports financial support was provided by 10.13039/501100000781European Research Council and Prof. Pedro P.C. de Souza reports that he was financed by the ERC/CONFAP/CNPq grant. If there are other authors, they declare that they have no known competing financial interests or personal relationships that could have appeared to influence the work reported in this paper.
