# Supplementary material for: Elucidating the mechano-molecular dynamics of TRAP activity using CRISPR/Cas9 mediated fluorescent reporter mice
Source: Heliyon. 2024 Jun 13;10(12):e32949. doi: 10.1016/j.heliyon.2024.e32949 (PMC11252717; doi:10.1016/j.heliyon.2024.e32949)
Supplement: Multimedia component 1 [file mmc1.docx]

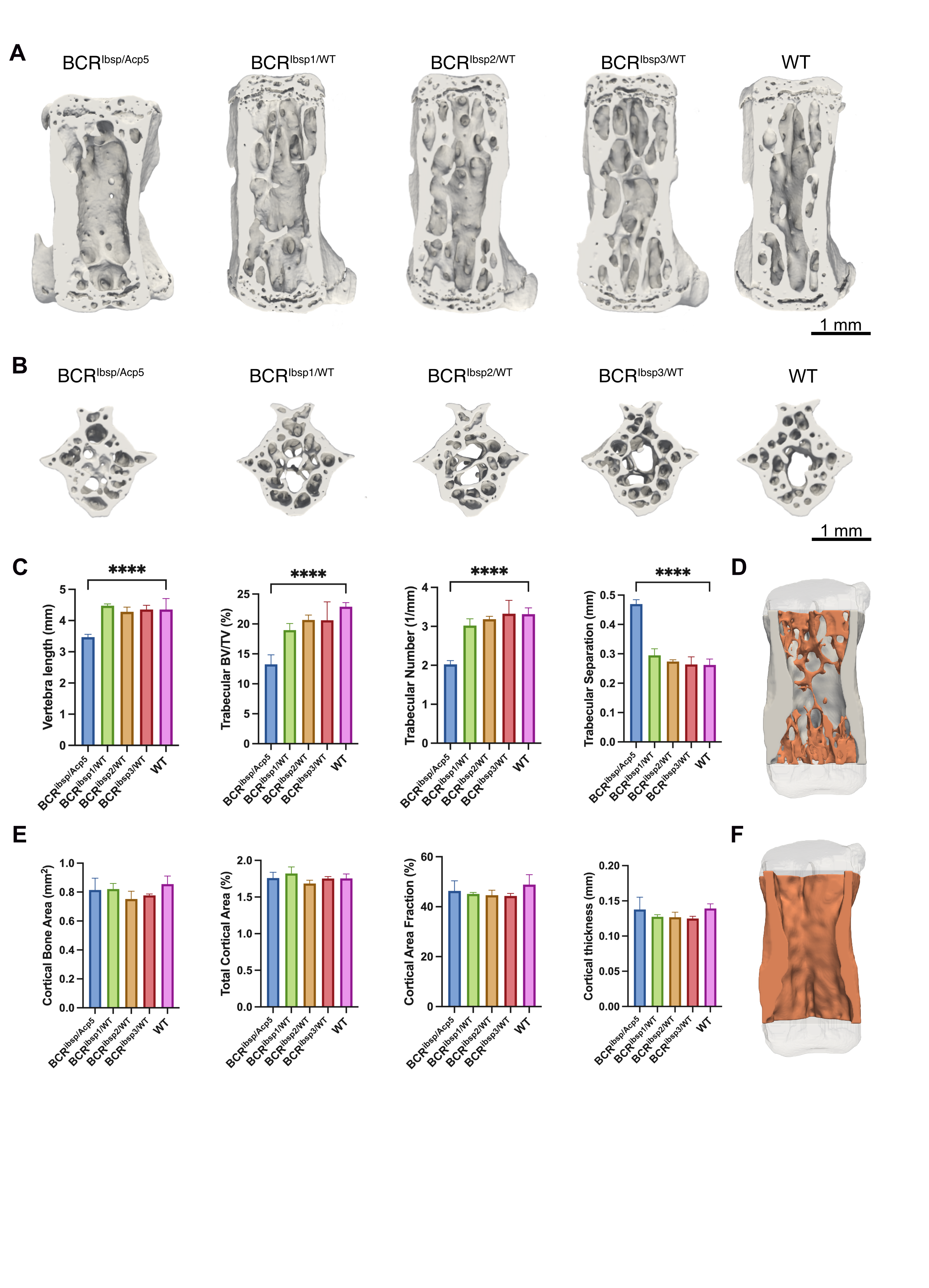


**Supplementary Figure 1 (Related to Figure 1): Micro-CT-based evaluation of caudal vertebra.** A) Representative 3D reconstructed longitudinal micro-CT images of the 6^th^ caudal vertebra of BCR^Ibsp/Acp5^, BCR^Ibsp/WT^, and WT mice at 20 weeks. B) Representative 3D reconstructed cross-sectional micro-CT images of the 6^th^ caudal vertebra of BCR^Ibsp/Acp5^, BCR^Ibsp/WT^, and WT mice at 20 weeks. C) Bone morphometric parameters obtained from micro-CT analysis; vertebra length, bone volume fraction (BV/TV), trabecular number, and trabecular separation. D) Representative 3D reconstructed longitudinal micro-CT images showing the trabecular mask in orange, where the analysis for trabecular parameters was obtained. E) Cortical bone parameters: cortical bone area, total cortical area, cortical area fraction, and cortical thickness. F) Representative 3D reconstructed longitudinal micro-CT images showing the cortical mask in orange where the analysis for cortical parameters was obtained.

Data represent mean ± s.d. (n=4-8 female mice/group), *<0.05, **<0.01, ***<0.001 according to one-way ANOVA with Dunnett test.


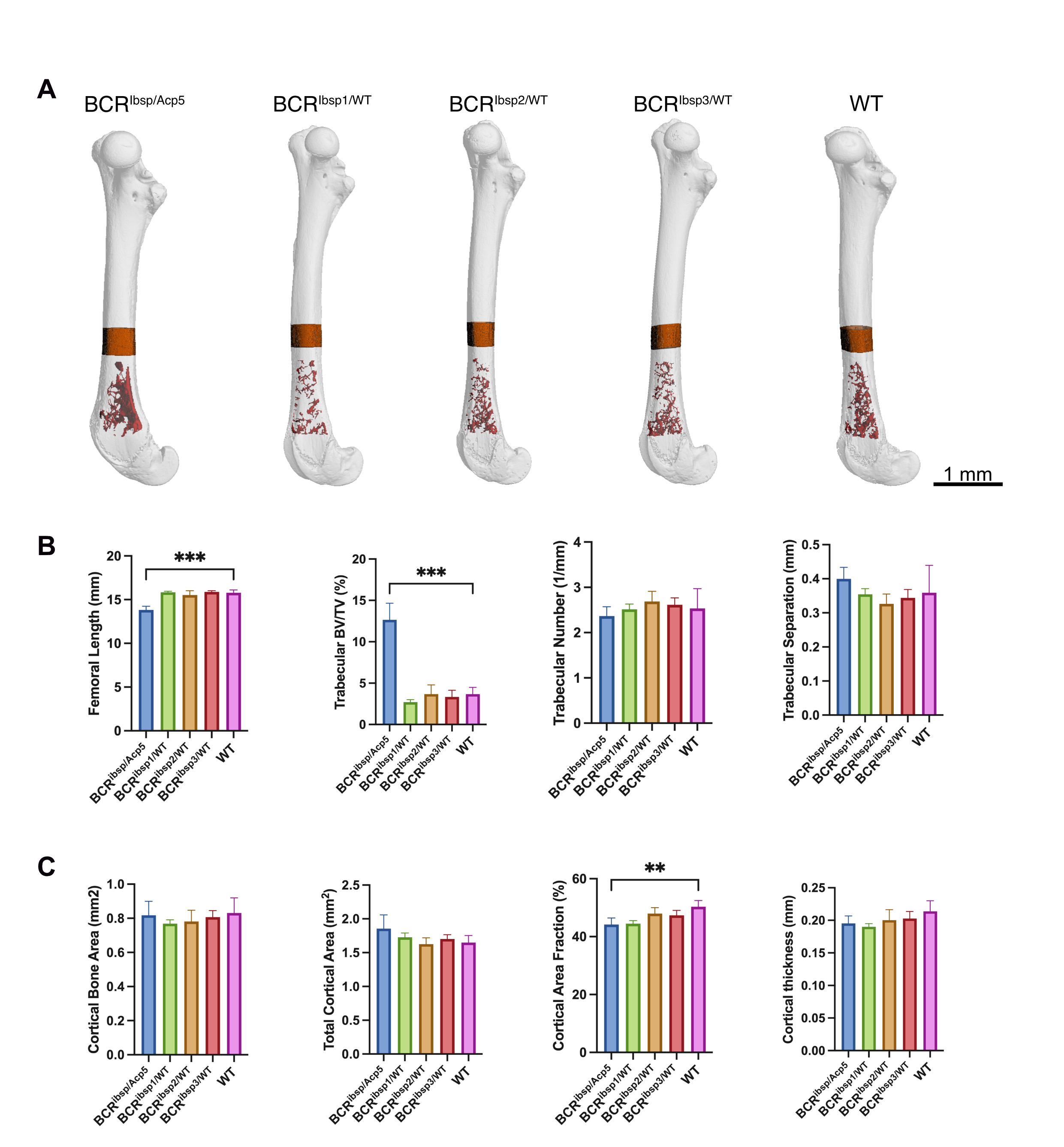


**Supplementary Figure 2 (Related to Figure 1): Micro-CT-based evaluation of femurs in BCR^Ibsp/Acp5^ mice.** A) Representative 3D reconstructed longitudinal micro-CT images of the femurs of BCR^Ibsp/Acp5^, BCR^Ibsp/WT^, and WT mice at 20 weeks. B) Bone morphometric parameters obtained from micro-CT analysis; femoral length, bone volume fraction (BV/TV), trabecular number, and trabecular separation. C) Cortical bone parameters: cortical bone area, total cortical area, cortical area fraction, and cortical thickness. Data represent mean ± s.d. (n=4-5 female mice/group), *<0.05, **<0.01, ***<0.001 according to one-way ANOVA with Dunnett test.


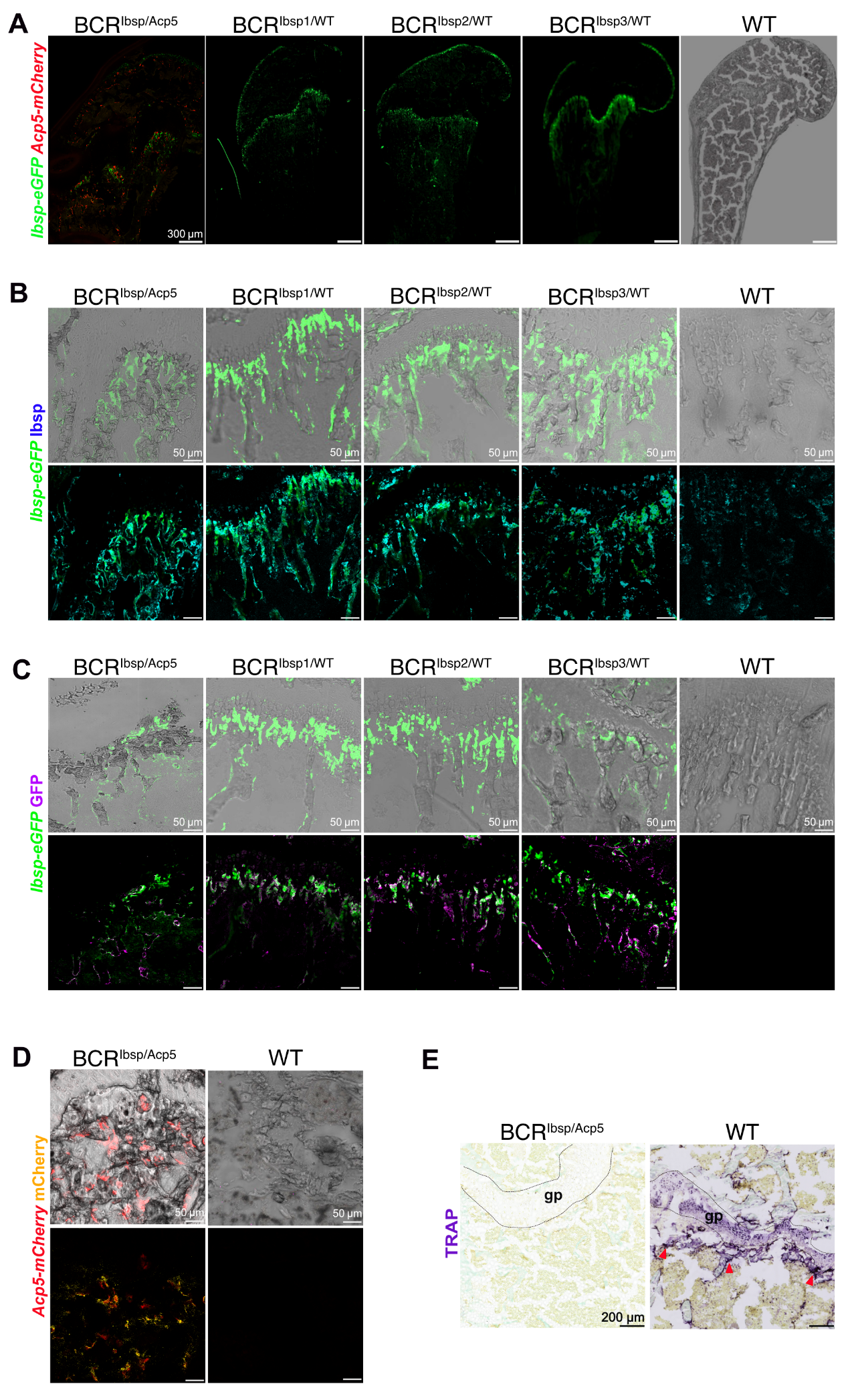


**Supplementary Figure 3 (Related to Figure 1): Morphological differences in the femurs of BCR^Ibsp/Acp5^ mice.** A) Representative tile scan confocal image of the right femur indicating GFP (*Ibsp-eGFP,* green) and mCherry (*Acp5-mCherry,* red) signals in female and/or male BCR^Ibsp/Acp5^**,** BCR^Ibsp/WT^, and WT mice. B) Colocalization of GFP (*Ibsp-eGFP*) signal with GFP immunostainings. GFP (*Ibsp-eGFP*, green) signal was shown on brightfield images for each staining. Representative maximum intensity projections images of BCR^Ibsp/Acp5^**,** BCR^Ibsp/WT^, and WT bones stained for GFP (purple) immunostaining. C) Colocalization of GFP (*Ibsp-eGFP*, green) with Ibsp immunostainings. GFP (*Ibsp-eGFP*, green) signal was demonstrated on brightfield images for each staining. Representative maximum intensity projections images of BCR^Ibsp/Acp5^**,** BCR^Ibsp/WT^, and WT bones stained for Ibsp (cyan) immunostaining. D) Colocalization of mCherry (*Acp5-mCherry*) signal with mCherry immunostainings. mCherry signal was indicated on the brightfield image. Representative maximum intensity projections images of BCR^Ibsp/Acp5^ and WT bones stained for mCherry (yellow) immunostaining. E) Histological images indicating TRAP (purple) staining around growth plate regions in BCR^Ibsp/Acp5^ and WT mice femurs.

**
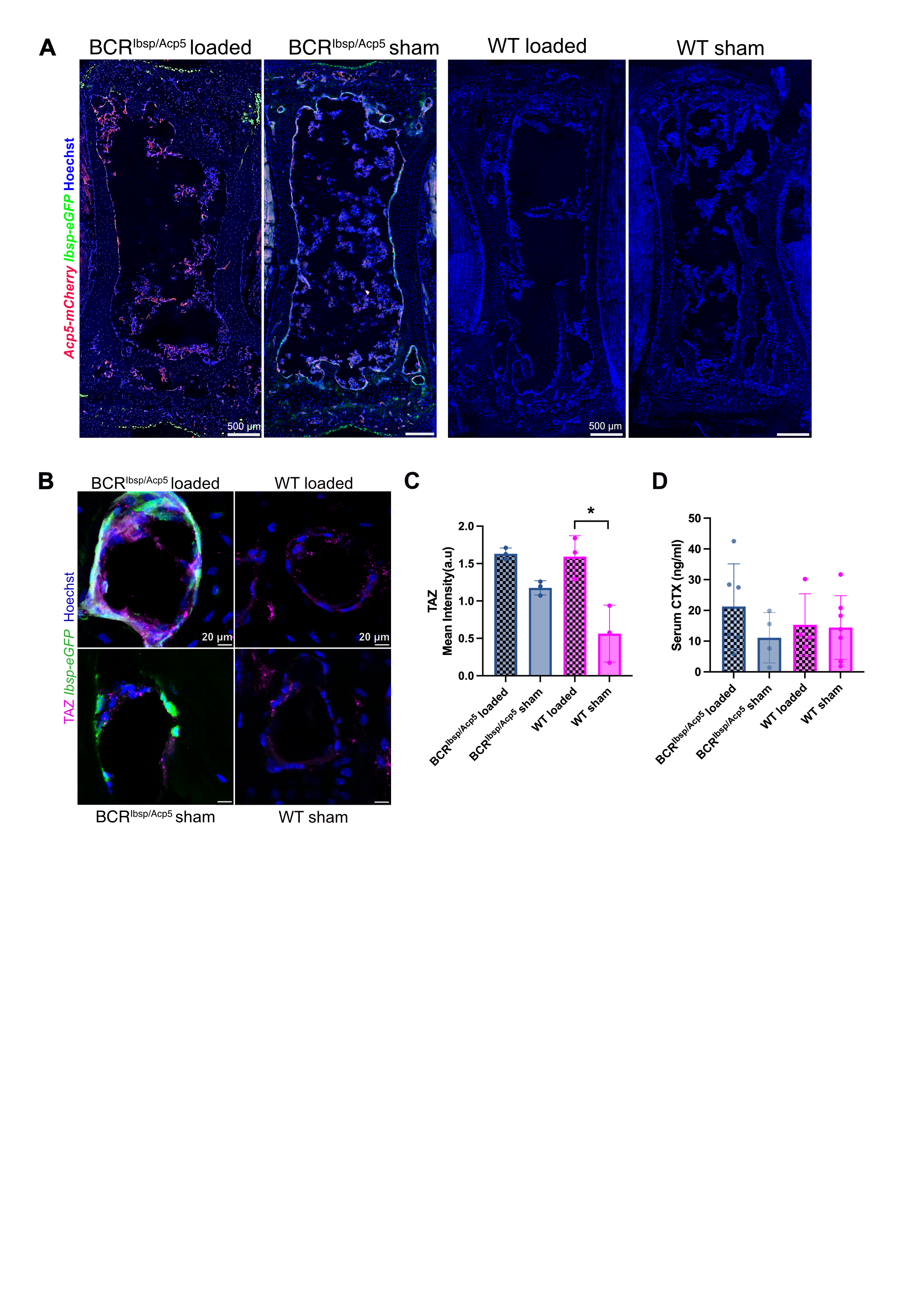
**

**Supplementary Figure 4 (Related to Figure 5): Mechano-molecular mechanism of BCR^Ibsp/Acp5^ mice *in vivo.*** A) Representative tile scan confocal images indicating mCherry (*Acp5-mCherry*, red), GFP (*Ibsp-eGFP*, green) signals and Hoechst (nucleus, blue) staining in loaded and sham-loaded BCR^Ibsp/Acp5^ and WT mice caudal vertebra. B) High-magnification images indicating GFP (*Ibsp-eGFP*, green) signal and TAZ staining (purple) in resorption cavities in loaded and sham-loaded BCR^Ibsp/Acp5^ and WT mice caudal vertebra sections. C) Mean intensity (a.u.) of TAZ signals from the resorption cavities from loaded and sham-loaded BCR^Ibsp/Acp5^ and WT mice. Data represent mean ± s.d., (n=3 female mice/group), *p<0.05, **p<0.01, ***p<0.001 according to one-way ANOVA with Tukey test. D) Measured serum CTX protein levels in loaded and sham-loaded BCR^Ibsp/Acp5^ and WT mice. Data represent mean ± s.d., (n= 4-7 female mice/group), *<0.05, **<0.01, ***<0.001 according to one-way ANOVA with Tukey test.


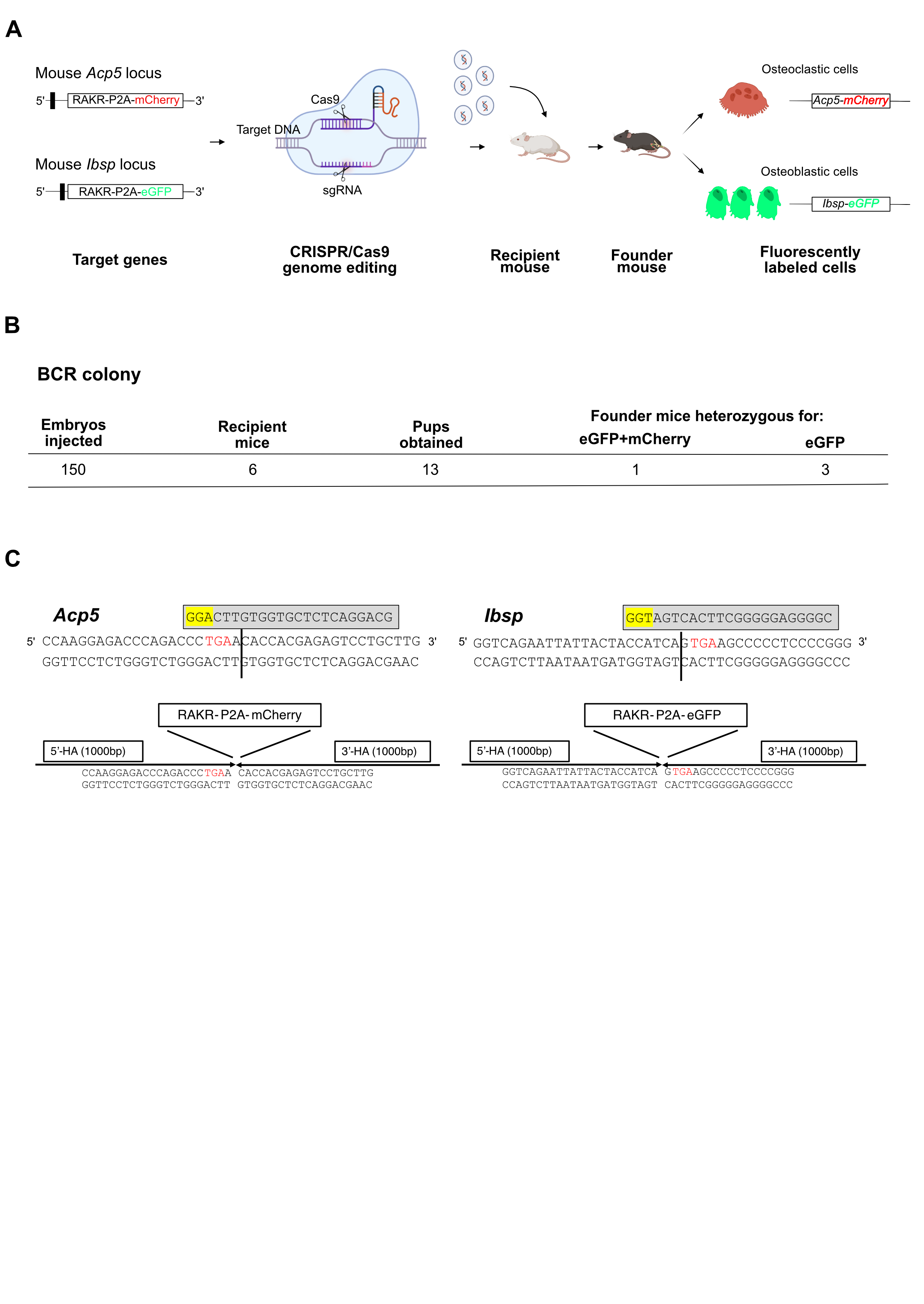
**Supplementary Figure 5 (Related to Figure 1): Generation of BCR mouse lines.** A) Schematic overview illustrating the targeting strategy for the *Acp5* and *Ibsp* locus. (created with Biorender). B) BCR mouse line colony information. C) Detailed targeting strategy in the *Acp5* and *Ibsp* locus for labeling osteoclastic and osteoblastic cells. Upper row: double-stranded target DNA with STOP codon (red), guide RNA (grey) including Protospacer Adjacent Motif (PAM) sequence (yellow), and Cas9 cutting site (black line). Lower row: DNA insertion site of donor templates (RAKR-P2A-mCherry; RAKR-P2A-eGFP) with an indication of homology arms (HA, 1000bp).

**Supplementary Table 1 (Related to Figure 1): Bone structural parameters in the full, trabecular, and cortical region of the 6^th^ caudal vertebra assessed by *ex vivo* micro-CT imaging**.
Parameter legend: total volume (TV), bone volume (BV), bone surface (BS), average volume density (AVD), bone surface density (BS/TV), specific bone surface (BS/BV); tissue mineral density (TMD), bone volume fraction (BV/TV), trabecular thickness (Tb.Th), trabecular number (Tb.N), trabecular separation (Tb.Sp), cortical area fraction (Ct.Ar/Tt.Ar), total cross-sectional area inside the periosteal envelope (Tt.Ar), cortical bone area (Ct.Ar), cortical thickness (Ct.Th). Data represent mean ± s.d. (n=4-8 female mice/group).

| **Parameters** | BCR^Ibsp/Acp5^ | BCR^Ibsp1/WT^ | BCR^Ibsp2/WT^ | BCR^Ibsp3/WT^ | WT |
| --- | --- | --- | --- | --- | --- |
| **Full** |  | | | | |
| TV [mm³] | 5.76±0.20 | 7.07±.0.25 | 6.35±0.25 | 6.61±0.08 | 6.60±0.36 |
| BV [mm³] | 3.44±0.29 | 4.14±0.17 | 3.74±0.27 | 3.84±0.12 | 4.18±0.25 |
| BS [mm²] | 53.24±1.52 | 82.53±2.20 | 76.04±3.21 | 80.82±2.96 | 72.48±4.60 |
| AVD [%] | 59.67±3.34 | 58.55±1.13 | 58.79±2.02 | 58.04±1.99 | 63.32±2.82 |
| BS/TV [1/mm] | 9.25±0.41 | 11.67±0.22 | 11.98±0.59 | 12.22±0.42 | 10.98±0.29 |
| BS/BV [1/mm] | 15.55±1.38 | 19.94±0.72 | 20.42±1.62 | 21.07±0.77 | 17.38±1.20 |
| TMD [mg HA/cm³] | 999.72±10.24 | 976.90±6.79 | 977.52±14.38 | 967.54±13.36 | 1002.24±7.06 |
| Length [mm] | 4.11±0.35 | 4.54±0.09 | 4.28±0.15 | 4.35±0.14 | 4.15±0.16 |
| **Trabecular** |  | | | | |
| BV/TV [%] | 13.26±1.57 | 18.96±1.11 | 20.68±0.80 | 20.61±3.09 | 23.70±1.93 |
| BS/TV [1/mm] | 4.38±0.46 | 6.27±0.17 | 6.91±0.29 | 6.97±0.73 | 6.84±0.39 |
| BS/BV [1/mm] | 33.37±4.78 | 33.09±1.30 | 33.46±2.29 | 34.00±1.99 | 28.99±2.65 |
| Tb.Sp [mm] | 0.47±0.02 | 0.29±0.02 | 0.27±0.01 | 0.26±0.03 | 0.26±0.02 |
| Tb.N [1/mm] | 2.03±0.10 | 3.02±0.17 | 3.19±0.07 | 3.19±0.07 | 3.32±0.34 |
| Tb.Th [mm] | 0.08±0.01 | 0.08±0.00 | 0.08±0.01 | 0.08±0.00 | 0.09±0.01 |
| **Cortical** |  | | | | |
| BS/TV [1/mm] | 4.61±0.32 | 5.41±0.16 | 5.53±0.28 | 5.64±0.23 | 5.40±0.24 |
| BS/BV [1/mm] | 10.01±1.02 | 11.99±0.50 | 12.44±1.03 | 12.72±0.51 | 11.10±0.83 |
| Ct.Ar/Tt.Ar [%] | 46.30±4.05 | 45.11±0.65 | 44.60±2.03 | 44.34±0.99 | 48.87±3.94 |
| Tt. Ar [%] | 0.81±0.08 | 0.82±0.04 | 0.75±0.05 | 0.78±0.01 | 1.65±0.10 |
| Ct. Ar [mm²] | 0.82±0.08 | 0.77±0.02 | 0.78±0.07 | 0.81±0.04 | 0.86±0.06 |
| Ct. Th [mm] | 0.14±0.02 | 0.13±0.00 | 0.13±0.01 | 0.12±0.00 | 0.14±0.01 |

**Supplementary Table 2 (Related to Figure 1): Bone structural parameters in the full, trabecular, and cortical regions of femurs assessed by *ex vivo* micro-CT imaging.**Parameter legend: total volume (TV), bone volume (BV), bone surface (BS), average volume density (AVD), bone surface density (BS/TV), specific bone surface (BS/BV); tissue mineral density (TMD), bone volume fraction (BV/TV), trabecular thickness (Tb.Th), trabecular number (Tb.N), trabecular separation (Tb.Sp), cortical area fraction (Ct.Ar/Tt.Ar), total cross-sectional area inside the periosteal envelope (Tt.Ar), cortical bone area (Ct.Ar), cortical thickness (Ct.Th). Data represent mean ± s.d., (n=4-5 female mice/group).

| **Parameters** | BCR^Ibsp/Acp5^ | BCR^Ibsp1/WT^ | BCR^Ibsp2/WT^ | BCR^Ibsp3/WT^ | WT |
| --- | --- | --- | --- | --- | --- |
| **Full** |  | | | | |
| TV [mm³] | 31.71±3.12 | 36.17±1.13 | 33.28±1.84 | 35.85±1.23 | 34.05±1.18 |
| BV [mm³] | 16.28±1.55 | 16.99±0.36 | 16.16±1.40 | 17.01±0.59 | 17.42±1.45 |
| BS [mm²] | 173.00±14.76 | 188.76±3.78 | 180.53±5.17 | 190.49±4.12 | 173.18±4.48 |
| AVD [%] | 51.36±1.00 | 46.97±0.94 | 48.51±1.70 | 47.46±0.91 | 51.10±2.65 |
| BS/TV [1/mm] | 5.46±0.18 | 5.22±0.10 | 5.43±0.24 | 5.32±0. 18 | 5.09±0.10 |
| BS/BV [1/mm] | 10.64±0.50 | 11.12±0.27 | 11.22±0.80 | 11.21±0.49 | 9.98±0.56 |
| TMD [mg HA/cm³] | 973.56±23.52 | 940.82±19.56 | 949.65±38.95 | 965.12±22.52 | 956.38±31.60 |
| Length [mm] | 13.83±0.40 | 15.84±0.12 | 15.53±0.48 | 15.89±0.14 | 15.76±0.31 |
| **Trabecular** |  | | | | |
| BV/TV [%] | 12.66±4.02 | 2.71±0.30 | 3.67±1.12 | 3.34±0.81 | 3.68±1.63 |
| BS/TV [1/mm] | 3.93±0.88 | 2.30±0.19 | 2.91±0.71 | 2.68±0.52 | 2.62±0.96 |
| BS/BV [1/mm] | 31.78±4.34 | 85.22±3.60 | 80.33±5.80 | 81.16±6.51 | 73.11±6.87 |
| Tb.Sp [mm] | 0.40±0.03 | 0.35±0.02 | 0.33±0.03 | 0.34±0.02 | 0.36±0.08 |
| Tb.N [1/mm] | 2.36±0.21 | 2.51±0.12 | 2.69±0.22 | 2.62±0.15 | 2.54±0.44 |
| Tb.Th [mm] | 0.10±0.01 | 0.04±0.00 | 0.04±0.00 | 0.04±0.00 | 0.05±0.00 |
| **Cortical** |  | | | | |
| BS/TV [1/mm] | 2.25±0.13 | 2.36±0.06 | 2.40±0.03 | 2.39±0.07 | 49.69±2.17 |
| BS/BV [1/mm] | 5.09±0.36 | 5.31±0.14 | 5.01±0.20 | 5.06±0.33 | 2.48±0.22 |
| Ct.Ar/Tt.Ar [%] | 44.18±2.23 | 44.47±1.05 | 47.99±2.04 | 47.40±1.70 | 4.94±0.53 |
| Tt. Ar [%] | 1.85±0.20 | 1.73±0.06 | 1.63±0.09 | 1.70±0.06 | 1.65±0.10 |
| Ct. Ar [mm²] | 0.82±0.08 | 0.77±0.02 | 0.78±0.07 | 0.81±0.04 | 0.83±0.09 |
| Ct. Th [mm] | 0.20±0.01 | 0.19±0.00 | 0.20±0.02 | 0.20±0.01 | 0.21±0.02 |

**Supplementary Table 3 (Related to Figure 2): Differentially expressed genes between BCR^Ibsp/Acp5^ and WT osteoclasts.** Differentially expressed genes are listed with FDR-adjusted p-value cutoff < 0.1. Data stored as Excel sheet (Supplementary Table 3.xlsx) in the Supplementary data section of the article.

**Supplementary Table 4 (Related to Figure 2): Gene ontology analysis for BCR^Ibsp/Acp5^ vs WT osteoclasts.** The significant GO terms were listed using an FDR-adjusted p-value < 0.01. Data stored as Excel sheet (Supplementary Table 4.xlsx) in the Supplementary data section of the article.

**Supplementary Table 5 (Related to Figure 4): Absolute values in the bone structural parameters in the trabecular and cortical region for the loaded and sham-loaded mice over the 4-week loading period.**

Parameter legend: trabecular bone volume fraction (BV/TV), trabecular thickness (Tb.Th), trabecular separation (Tb.Sp), trabecular number (Tb.N), tissue mineral density (TMD), cortical area fraction (Ct.Ar/Tt.Ar), cortical bone area (Ct.Ar) and cortical thickness (Ct.Th). Data represent mean ± s.d., (n=8-10 female mice/group)

| **Parameter** | **BCR^Ibsp/Acp5^ loaded** | **BCR^Ibsp/Acp5^ sham-loaded** | **WT loaded** | **WT sham-loaded** |
| --- | --- | --- | --- | --- |
| **Trabecular** |  | | | |
| **BV/TV [%]**  Week 0  Week 1  Week 2  Week 3  Week 4 | 10.64±1.08  11.92±1.30  12.92±1.32  13.70±1.76  14.60±1.64 | 13.44±2.83  13.41±2.81  13.08±2.80  13.05±2.94  13.00±2.92 | 16.48±2.57  17.51±2.02  18.12±1.93  19.14±1.90  19.88±2.08 | 15.92±1.87  15.78±2.29  15.68±2.57  15.73±2.80  15.76±2.77 |
| **Tb.Th [mm]**  Week 0  Week 1  Week 2  Week 3  Week 4 | 0.07±0.00  0.08±0.00  0.09±0.00  0.10±0.01  0.11±0.01 | 0.08±0.00  0.08±0.00  0.08±0.00  0.08±0.00  0.08±0.00 | 0.07±0.00  0.07±0.00  0.08±0.00  0.08±0.00  0.09±0.00 | 0.06±0.00  0.07±0.00  0.07±0.00  0.07±0.00  0.07±0.00 |
| **Tb.Sp [mm]**  Week 0  Week 1  Week 2  Week 3  Week 4 | 0.49±0.04  0.50±0.05  0.50±0.04  0.50±0.05  0.50±0.05 | 0.46±0.04  0.46±0.05  0.47±0.04  0.48±0.06  0.48±0.06 | 0.32±0.03  0.32±0.04  0.33±0.04  0.33±0.04  0.33±0.04 | 0.31±0.02  0.31±0.02  0.32±0.03  0.33±0.04  0.33±0.04 |
| **Tb.N [1/mm]**  Week 0  Week 1  Week 2  Week 3  Week 4 | 1.92±0.16  1.89±0.18  1.86±0.18  1.84±0.18  1.82±0.17 | 2.10±0.22  2.04±0.24  2.02±0.23  1.99±0.26  1.97±0.24 | 2.85±0.24  2.79±0.27  2.76±0.24  2.73±0.26  2.69±0.24 | 2.98±0.17  2.90±0.18  2.83±0.23  2.81±0.23  2.76±0.25 |
| **TMD [mg HA/cm³]**  Week 0  Week 1  Week 2  Week 3  Week 4 | 1094.92±5.94  1101.52±5.30  1103.81±7.20  1110.64±4.96  1116.03±5.42 | 1093.39±7.13  1098.16±5.59  1100.06±4.41  1105.66±4.99  1108.10±3.87 | 1100.36±4.79  1101.08±6.19  1104.80±5.03  1106.66±7.20  1116.34±4.03 | 1097.51±9.06  1100.67±7.94  1105.41±6.38  1105.26±5.88  1105.54±8.30 |
| **Cortical** |  | | | |
| **Ct.Ar/Tt.Ar [%]**  Week 0  Week 1  Week 2  Week 3  Week 4 | 43.56±1.78  44.35±1.69  45.62±1.75  47.18±1.65  48.32±1.75 | 42.49±2.51  42.58±2.76  42.54±2.68  42.52±2.64  42.48±2.63 | 37.23±2.09  38.32±2.05  40.14±2.10  41.30±2.25  42.34±2.12 | 35.05±2.79  36.18±2.83  36.55±2.87  36.85±2.79  37.09±2.73 |
| **Ct.Ar [mm²]**  Week 0  Week 1  Week 2  Week 3  Week 4 | 0.78±0.05  0.80±0.04  0.82±0.05  0.86±0.04  0.88±0.03 | 0.78±0.05  0.79±0.05  0.79±0.05  0.80±0.04  0.80±0.04 | 0.68±0.02  0.71±0.03  0.74±0.03  0.77±0.03  0.79±0.03 | 0.66±0.06  0.66±0.06  0.68±0.06  0.68±0.06  0.69±0.06 |
| **Ct.Th [mm]**  Week 0  Week 1  Week 2  Week 3  Week 4 | 0.16±0.01  0.16±0.01  0.16±0.01  0.17±0.01  0.18±0.01 | 0.15±0.01  0.15±0.01  0.15±0.01  0.15±0.01  0.15±0.01 | 0.13±0.01  0.14±0.01  0.14±0.01  0.15±0.01  0.15±0.01 | 0.13±0.01  0.13±0.01  0.13±0.01  0.13±0.01  0.13±0.01 |

**Supplementary Table 6 (Related to Figure 6)**: Parameters of the mathematical functions fitted to the estimated mechanostat group average remodeling velocity curves for all weekly intervals. Data presented as “parameter (95% confidence interval)”, where the 95% confidence interval was calculated using the balanced bias-corrected and accelerated bootstrapping approach, as described in the methods section. Normalized root mean squared error (NRMSE) was used to characterize the quality of the fit, considering the range defined by the minimum and maximum observed RmV values as the normalization parameter. The row “Effective strain range” indicates the range of mechanical signal values from which the fit of the mathematical functions was derived. (n=9-10 female mice/group).

Parameter legend: Resorption saturation level (RSL), Resorption velocity modulus (RVM), Resorption threshold (RT), Formation threshold (FT), Formation velocity modulus (FVM), Formation saturation level (FSL), (Re)modeling threshold (RmT), (Re)modeling velocity modulus (RmVM).

| Weeks | Parameter | Unit | Group | | | |
| --- | --- | --- | --- | --- | --- | --- |
|  |  |  | BCR^Ibsp/Acp5^ sham | BCR^Ibsp/Acp5^ loaded | WT sham | WT loaded |
| 0-1 | RSL | µm/day | -3.130 (-4.679, -1.445) | -1.388 (-1.844, -0.480) | -1.923 (-2.684, -0.692) | -0.729 (-1.211, -0.315) |
|  | RmVM | (µm/day) x µε | 114 (52, 152) | 384 (170, 543) | 185 (73, 282) | 568 (144, 832) |
|  | RmT | µε | 386 (303, 723) | 227 (191, 326) | 363 (301, 881) | 289 (265, 352) |
|  | FSL | µm/day | 0.202 (0.154, 0.367) | 0.897 (0.834, 1.062) | 0.283 (0.173, 0.594) | 0.682 (0.594, 0.855) |
|  | NRMSE | % | 2.75 (2.40, 5.13) | 3.94 (3.84, 6.56) | 3.74 (3.43, 7.03) | 3.79 (3.57, 6.43) |
|  | Effective strain range | µε | 10–1470 | 10–2950 | 10–1210 | 10–2180 |
| 1-2 | RSL | µm/day | -3.750 (-4.272, -2.127) | -1.506 (-2.020, -0.248) | -2.799 (-4.013, -0.924) | -0.985 (-1.645, -0.327) |
|  | RmVM | (µm/day) x µε | 115 (81, 137) | 318 (183, 376) | 131 (35, 165) | 228 (114, 334) |
|  | RmT | µε | 670 (534, 5040) | 168 (136, 270) | 357 (288, 11534) | 184 (169, 219) |
|  | FSL | µm/day | 0.087 (0.042, 0.199) | 1.035 (0.954, 1.264) | 0.227 (0.058, 0.564) | 0.633 (0.576, 0.807) |
|  | NRMSE | % | 1.81 (1.81, 4.15) | 3.86 (3.72, 6.46) | 4.10 (3.49, 6.99) | 3.10 (3.13, 5.46) |
|  | Effective strain range | µε | 10–1430 | 10–2800 | 0–1250 | 10–1900 |
| 2-3 | RSL | µm/day | -2.440 (-2.950, -1.235) | -0.180 (-0.715, 0.428) | -1.186 (-1.736, -0.410) | -2.794 (-3.898, -0.725) |
|  | RmVM | (µm/day) x µε | 111 (64, 133) | 472 (75, 1120) | 284 (87, 452) | 118 (65, 133) |
|  | RmT | µε | 421 (378, 776) | 66 (0, 157) | 363 (319, 554) | 194 (167, 253) |
|  | FSL | µm/day | 0.169 (0.125, 0.292) | 0.985 (0.892, 1.489) | 0.328 (0.281, 0.410) | 0.472 (0.452, 0.538) |
|  | NRMSE | % | 2.17 (2.03, 4.60) | 5.08 (4.67, 11.27) | 3.81 (3.15, 9.22) | 2.17 (1.83, 5.05) |
|  | Effective strain range | µε | 10–1430 | 10–2500 | 0–1190 | 10–1740 |
| 3-4 | RSL | µm/day | -2.347 (-2.784, -1.262) | -0.949 (-1.469, 0.015) | -1.362 (-1.863, -0.654) | -1.521 (-2.284, -0.563) |
|  | RmVM | (µm/day) x µε | 150 (105, 177) | 186 (72, 281) | 245 (61, 337) | 283 (105, 415) |
|  | RmT | µε | 366 (340, 483) | 122 (98, 164) | 335 (306, 434) | 286 (254, 361) |
|  | FSL | µm/day | 0.263 (0.220, 0.365) | 0.786 (0.749, 0.912) | 0.334 (0.276, 0.453) | 0.542 (0.475, 0.689) |
|  | NRMSE | % | 2.27 (2.27, 3.94) | 3.93 (3.28, 7.23) | 2.84 (2.70, 5.42) | 3.02 (2.86, 6.33) |
|  | Effective strain range | µε | 10–1430 | 10–2210 | 0–1140 | 10–1660 |

**Supplementary Table 7 (Related to Figure 6):** Statistical outcomes (p-values) of the pair comparisons of parameters obtained from the hyperbola functions fitted to the RmV curves, for each week and group pair. The significance values were adjusted for multiple comparisons using Bonferroni correction. These comparisons considered the parameters fitted to the average curves and parameter distributions estimated using the balanced bias-corrected and accelerated bootstrapping approach described in the methods. (n=9-10 female mice/group).

Parameter legend: Resorption saturation level (RSL), Resorption velocity modulus (RVM), Resorption threshold (RT), Formation threshold (FT), Formation velocity modulus (FVM), Formation saturation level (FSL), (Re)modeling threshold (RmT), (Re)modeling velocity modulus (RmVM). Significant outcomes at a level of 0.05 are highlighted in bold.

| Weeks | Group comparison | Parameter | | | |
| --- | --- | --- | --- | --- | --- |
|  |  | RSL | RmVM | RmT | FSL |
| 0-1 | BCR^Ibsp/Acp5^ sham – BCR^Ibsp/Acp5^ loaded | 0.29200 | **0.00109** | **0.03433** | **0.00001** |
|  | BCR^Ibsp/Acp5^ sham – WT sham | 0.98784 | 0.06902 | 0.43463 | 0.81011 |
|  | BCR^Ibsp/Acp5^ sham – WT loaded | 0.14340 | **0.00119** | 0.07198 | **0.00660** |
|  | BCR^Ibsp/Acp5^ loaded – WT sham | 0.04999 | 0.91072 | 0.82559 | **0.00001** |
|  | BCR^Ibsp/Acp5^ loaded – WT loaded | 0.84749 | 0.11589 | 0.26757 | **0.00073** |
|  | WT sham – WT loaded | 0.64182 | **0.01775** | 0.08072 | **0.02298** |
| 1-2 | BCR^Ibsp/Acp5^ sham – BCR^Ibsp/Acp5^ loaded | **0.03813** | **0.00001** | **0.00001** | **0.00001** |
|  | BCR^Ibsp/Acp5^ sham – WT sham | 0.79458 | 0.14307 | 0.11353 | 0.96187 |
|  | BCR^Ibsp/Acp5^ sham – WT loaded | **0.00001** | **0.00001** | **0.00001** | **0.00001** |
|  | BCR^Ibsp/Acp5^ loaded – WT sham | **0.01934** | 0.50233 | 0.93048 | **0.00001** |
|  | BCR^Ibsp/Acp5^ loaded – WT loaded | 0.78424 | 0.78935 | 0.54723 | **0.00001** |
|  | WT sham – WT loaded | 0.22901 | **0.03181** | **0.01932** | **0.00001** |
| 2-3 | BCR^Ibsp/Acp5^ sham – BCR^Ibsp/Acp5^ loaded | **0.02657** | **0.00907** | **0.00001** | **0.00001** |
|  | BCR^Ibsp/Acp5^ sham – WT sham | 0.60730 | **0.00145** | 0.32990 | 0.10227 |
|  | BCR^Ibsp/Acp5^ sham – WT loaded | 0.16744 | 0.20277 | **0.00001** | **0.00001** |
|  | BCR^Ibsp/Acp5^ loaded – WT sham | **0.02519** | 0.70416 | **0.00001** | **0.00001** |
|  | BCR^Ibsp/Acp5^ loaded – WT loaded | **0.00026** | 0.51138 | 0.15333 | **0.00001** |
|  | WT sham – WT loaded | **0.01028** | 0.44383 | **0.00027** | **0.00001** |
| 3-4 | BCR^Ibsp/Acp5^ sham – BCR^Ibsp/Acp5^ loaded | 0.23250 | 0.13044 | **0.00001** | **0.00001** |
|  | BCR^Ibsp/Acp5^ sham – WT sham | 0.58683 | **0.04209** | 0.58523 | 0.87816 |
|  | BCR^Ibsp/Acp5^ sham – WT loaded | 0.85009 | **0.02283** | **0.00441** | **0.02986** |
|  | BCR^Ibsp/Acp5^ loaded – WT sham | 0.11580 | 0.12690 | **0.00001** | **0.00001** |
|  | BCR^Ibsp/Acp5^ loaded – WT loaded | 0.06675 | 0.10081 | **0.00001** | **0.00001** |
|  | WT sham – WT loaded | 0.18647 | 0.19926 | 0.05884 | 0.39030 |
